# Seasonal trends of nutrient intake in rainforest communities of north-eastern Madagascar

**DOI:** 10.1017/S1368980019001083

**Published:** 2019-08

**Authors:** Christopher D Golden, Bapu Vaitla, Laurent Ravaoliny, Miadana A Vonona, EJ Gasta Anjaranirina, Hervet J Randriamady, Raymond P Glahn, Sarah E Guth, Lia CH Fernald, Samuel S Myers

**Affiliations:** 1 Harvard T.H. Chan School of Public Health, Department of Environmental Health, Boston, MA, USA; 2 Harvard University Center for the Environment, 26 Oxford Street 4th Floor, Cambridge, MA 02138, USA; 3 Madagascar Health and Environmental Research (MAHERY), Maroantsetra, Madagascar; 4 Cornell University, Department of Food Science, Ithaca, NY, USA; 5 University of California at Berkeley, School of Public Health, Berkeley, CA, USA

**Keywords:** Dietary intake, Food security, Micronutrients, Planetary health, Wild foods, Animal-source foods

## Abstract

**Objective:**

We collected dietary records over the course of nine months to comprehensively characterize the consumption patterns of Malagasy people living in remote rainforest areas of north-eastern Madagascar.

**Design:**

The present study was a prospective longitudinal cohort study to estimate dietary diversity and nutrient intake for a suite of macronutrients, micronutrients and vitamins for 152 randomly selected households in two communities.

**Setting:**

Madagascar, with over 25 million people living in an area the size of France, faces a multitude of nutritional challenges. Micronutrient-poor staples, especially rice, roots and tubers, comprise nearly 80 % of the Malagasy diet by weight. The remaining dietary components (including wild foods and animal-source foods) are critical for nutrition. We focus our study in north-eastern Madagascar, characterized by access to rainforest, rice paddies and local agriculture.

**Participants:**

We enrolled men, women and children of both sexes and all ages in a randomized sample of households in two communities.

**Results:**

Although the Household Dietary Diversity Score and Food Consumption Score reflect high dietary diversity, the Minimum Dietary Diversity–Women indicator suggests poor micronutrient adequacy. The food intake data confirm a mixed nutritional picture. We found that the median individual consumed less than 50 % of his/her age/sex-specific Estimated Average Requirement (EAR) for vitamins A, B_12_, D and E, and Ca, and less than 100 % of his/her EAR for energy, riboflavin, folate and Na.

**Conclusions:**

Malnutrition in remote communities of north-eastern Madagascar is pervasive and multidimensional, indicating an urgent need for comprehensive public health and development interventions focused on providing nutritional security.

Madagascar, with a population of over 25 million living in an area the size of France and growing by 3 % per year^(^^1^^)^, faces a multitude of nutritional challenges. The country contains many regions of stark food scarcity and, at the national level, the population is one of the most chronically undernourished in the world, with nearly 50 % of children under 5 years of age stunted^(^^2^^)^. With 78 % of the population living in poverty^(^^2^^)^ and 64 % of the population thinly dispersed in rural (and often remote) areas^(^^3^^)^, the barriers to improving the affordability and quality of diets through national and local to global markets are considerable.

The food system in Madagascar is structured around rice. Malagasy people eat approximately 300 g of rice (dry weight) per person per day, more than nearly any other country^(^^4^^)^. In addition to rice, the Malagasy diet varies regionally but largely consists of starchy tubers (cassava, yam, sweet potato, taro), maize, plantains, and a variety of greens and other vegetables. Seafood is an animal-source food staple along the coast, while chickens, ducks, pigs, goats and zebu provide animal-source foods inland. Outside agricultural systems, people throughout the country are heavily reliant on wild foods for nutrition, including mammalian wildlife, insects, seafood, and wild fruit and vegetables^(^^5^^–^^8^^)^. These wild foods are critically important in increasing dietary diversity, especially given the scarcity and cost of animal-source foods, high-starch diets with low micronutrient content, and the potential for extreme weather events, climate change and disease to disrupt agroecosystems. These challenges are even more important to understand in the context of remote rainforest areas of north-eastern Madagascar where reliance on wild foods is much higher than other parts of the country^(^^9^^)^ and where seasonality will likely play a large role in determining the quantity and quality of foods available. By characterizing the diet in these remote regions, policy makers and public health practitioners can best identify foods that are culturally and nutritionally important, and target interventions to reduce rates of malnutrition of similar populations.

Dietary intake is commonly evaluated by assessment tools like FFQ, dietary diversity scores and 24 h recalls. These tools are used to produce composite indicators of food consumption, including the Household Dietary Diversity Score (HDDS), Food Consumption Score (FCS) and Minimum Dietary Diversity for Women (MDD-W), or to undertake more direct nutrient intake calculations to characterize household food access and to determine the adequacy and quality of diets. However, data for these indicators are usually collected once or at multiple points over time, on the assumption that dietary patterns on the day(s) can provide insight into the diet across seasons and years^(^^10^^)^. In the context of the highly seasonal environment of Madagascar, such an assumption is likely to lead to inaccurate estimates of food consumption and nutritional status. Moreover, FCS and HDDS have not been well validated for diet quality and other individual-level indicators would be required to better understand diet quality^(^^11^^)^. In contexts that are heavily reliant on biodiverse and seasonal foods (such as the case here), dietary species richness may be an important factor to consider, although it is beyond the scope of the current study^(^^12^^)^. We thus use detailed daily dietary records (data on three meals per day, every day, for nine consecutive months) to evaluate current nutrient intake and assess dietary inadequacies in two communities.

## Methods

### Study site

Madagascar is characterized by highly variable climates and ecosystems, ranging from desert conditions in the south-western parts of the country to some of the wettest geographies on earth in the eastern rainforests. These local microclimates have major implications on the food production systems characteristic of each region and influence food availability seasonally within a given geography^(^^13^^)^. Given the lack of well-functioning markets in Madagascar^(^^14^^)^, environmental conditions thus play a critical role in determining food availability and access.

**Fig. 1:**
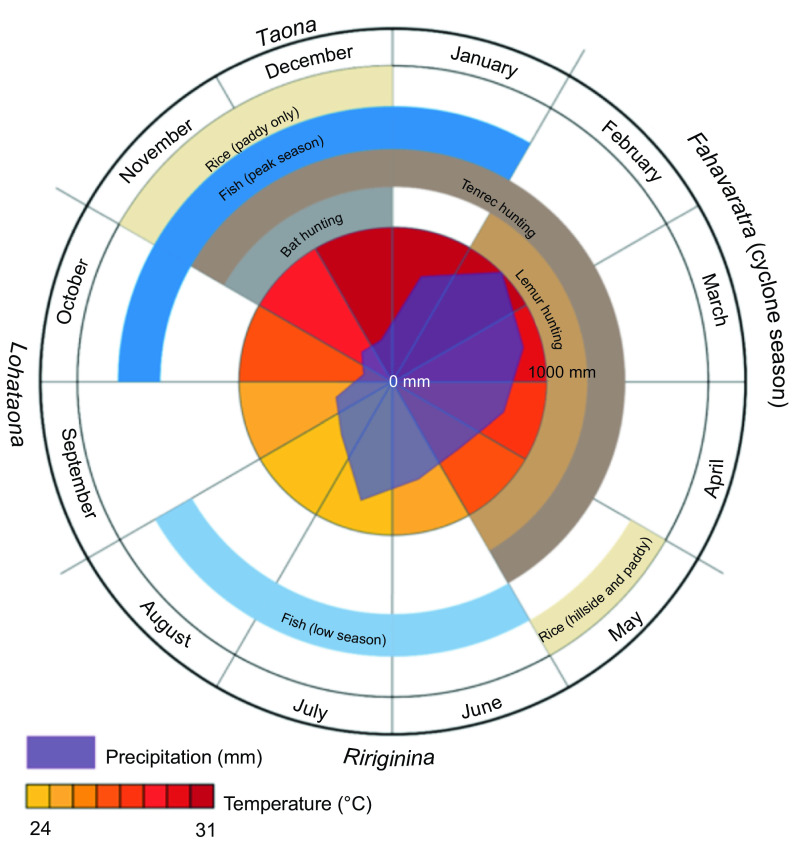
Seasons in Madagascar recognized by the local Malagasy in the Maroantsetra region

Although Madagascar broadly has two seasons (cold/dry and hot/wet), the local Malagasy in the Maroantsetra region recognize four seasons: *ririginina* (cold/wet), *lohataona* (hot/dry); *taona* (hot/wet); and *fahavaratra* (cyclone season; Fig. 1). The Makira Natural Park is located in north-eastern Madagascar, west of the city of Maroantsetra, where consistent rains throughout the year and frequent cyclones make many types of conventional agricultural production difficult^(^^15^^)^. Most non-native, high-volume vegetable production (e.g. cabbage, carrots, onions, etc.) is centred in the cooler, drier climates of the High Plateau. However, the north-east is one of the largest rice production centres in the country because of its abundant access to surface water without irrigation. The communities adjacent to Makira Natural Park are almost exclusively comprised of agriculturalists, with a focus on rice and tuber production and a suite of cash crops (especially vanilla, cloves and coffee). The severe poverty in this region, coupled with a weak transport infrastructure, leads to almost total autarchic food production systems based on small-scale, low-input agriculture and a heavy reliance on wild foods.

### Data collection

From January 2013 to April 2014, we followed a cohort of individuals from two communities in north-eastern Madagascar where the research team had spent nearly a decade conducting research. Within these two communities, there were 152 randomly sampled households that were tracked to understand the dietary determinants of nutritional status in Malagasy men, women and children. The sample included 719 individuals of both sexes, spanning in age from newborn to 73 years, in two communities adjacent to the Makira Natural Park^(^^16^^)^.

Data collection included daily household diet records and monthly assessment of socio-economic status, natural resource use, self-reported illness and treatment, and self-reported consumption of food outside the household. Each female head of household recorded the weight (to the nearest gram) of all food that was cooked in the household for three meals per day every day for the duration of the study, with the final data set including nearly 250 distinct foods. Clinically, we collected information at three distinct time periods (each four to five months apart) on individual anthropometry, micronutrient status (Hb, vitamin A, vitamin B_12_, Fe, Zn, ferritin, transferrin receptor), inflammation levels (C-reactive protein and α_1_-acid glycoprotein), and the presence of malaria, intestinal parasites and other types of disease. For extensive details on all protocols and procedures of this research study, please refer to the MAHERY study cohort profile^(^^16^^)^.

We found that the data quality of household food measurement improved considerably after an initial learning period (determined by adherence to data recording and frequency of outliers) and so, for the purposes of the current analysis, we utilize only the highest-quality data spanning the nine-month time frame from July 2013 to March 2014. The beginning of this period corresponds roughly to the time of the first clinical data collection, and the end of the period to the third and final clinical data collection. We then examined this data set for meal weight outliers and missing meal records. We divided outliers into two groups, improbably high values (0·4 % of all household-days) and improbably low values (11·5 % of all household-days). Given the lack of pre-existing quantitative food consumption data on this population by which to make distributional assumptions, we chose outlier thresholds manually: high outliers are those days in which per capita food consumption exceeded 5 kg; low outliers are those days in which per capita food consumption was below 500 g or energy intake was less than 30 % of the household’s Estimated Average Requirement (EAR). For all outlier and missing days, we replaced such values with reconstructed diets using household-specific means over the entire time series for each specific food.

Prior research by the same team in the same population suggests that everyone present in the household for a meal ate all the types of food available for that meal, with rare exception^(^^17^^)^. That research also found that the weight of food consumed at household meals by individuals is in proportion to each person’s relative body weight^(^^17^^)^. For all individuals above 6 months old, we thus assigned food intake from household meals according to this evidence. Infants under 6 months old generally consume minimal amounts of complementary foods in this population and thus were excluded from this partitioning of household meals. We assumed that all members of the household consumed all foods reported at common meals. We thus created an individual-level data set of foods consumed within the household. We then added individual-level data on foods consumed outside the household, as reported by each individual. These out-of-household data were reported on a monthly basis, with frequency of consumption during the month recalled by the respondent. In order to harmonize with the within-household daily data, we distributed foods appearing in the monthly out-of-household data randomly across the days within the month, with the number of days corresponding to the frequency of reported consumption. Finally, we combined the within-household data and the out-of-household data to calculate individual daily dietary intake. Because the weight of food consumed varies considerably day to day (influenced by wild meat capture, feasts and other factors), we present nutrient adequacy results by month. Table 1 below thus shows mean and median nutrient intake sufficiency taken over all calculations of monthly individual intakes.

**Table 1:** The mean and median nutrient intake sufficiency in the diet of 719 individuals of both sexes and all ages, north-eastern Madagascar, July 2013 to March 2014

Nutrient	Mean adequacy (% of EAR)	sd	Median adequacy (% of EAR)	Median status*
Energy	69·9	28·3	65·6	Inadequate
Protein	150·9	68·2	135·4	Adequate
Carbohydrates	264·4	137·4	255·9	Adequate
Vitamin A	51·7	54·4	36·5	Severely inadequate
Thiamin	129·7	77·9	109·3	Adequate
Riboflavin	72·3	39·0	63·6	Inadequate
Niacin	136·5	54·3	129·0	Adequate
Vitamin B_6_	174·2	92·3	152·0	Adequate
Folate	72·9	38·2	64·7	Inadequate
Vitamin B_12_	50·3	57·7	35·5	Severely inadequate
Vitamin C	233·7	322·8	156·0	Adequate
Vitamin D	14·3	25·3	7·5	Severely inadequate
Vitamin E	40·2	27·3	33·3	Severely inadequate
Ca	25·2	17·6	20·8	Severely inadequate
Cu	280·3	124·1	255·4	Adequate
Fe	146·7	70·3	131·9	Adequate
Mg	205·0	78·4	192·3	Adequate
P	153·6	82·8	140·4	Adequate
Na	102·9	79·2	83·8	Inadequate
Zn	126·7	55·3	114·5	Adequate

EAR, Estimated Average Requirement.

* We compared each calculated monthly individual nutrient intake with the respective individual’s EAR, thus obtaining adequacy (intake as % of EAR) values for each monthly individual calculated intake. The mean and median adequacy values above are drawn from this distribution of adequacy values. Median status was defined as adequate (≥100 % of EAR), inadequate (≥50 to <100 % of EAR) or severely inadequate (<50 % of EAR).

We compiled a nutrient composition database of the 250 foods included in the daily household diet records using existing online food composition tables. We collected nutritional information for 100 g servings of each food, prioritizing nutritional information from African databases and then filling in missing micronutrient data with additional sources (see online supplementary material, Supplemental Table S1). We proxied nutritional information for local food varieties by using data from closely related varieties most often found in African databases, although we occasionally proxied seafood using Asian and American databases. When nutritional information for local varieties was unavailable, we used the US Department of Agriculture food composition database. For example, we proxied the local variety of nightshade (*Solanum erthracanthum*) with Ethiopian nightshade (*Solanum aethiopicum*); however, because we were unable to find nutritional information for bush meat closely related to the African bushpig (*Potamochoerus larvatus*), we used wild boar meat (from the US Department of Agriculture database) as a proxy. When we were unable to identify appropriate proxy foods for endemic Malagasy foods (e.g. local fruits), we calculated weighted averages of micronutrient data from food items in the same category (food categories are listed in Supplemental Table S2). We weighted averages by the proportion of the daily consumption of each food item to the total daily consumption of food items in the group being averaged. We then multiplied daily food quantities by these nutrient values to obtain daily household nutrient intake.

In synthesizing nutritional information from existing online databases, we were able to compile a complete list of micronutrient data for a wide variety of foods. However, compiling data from such diversity in sources may have produced inconsistencies. Some sources specified how food items were prepared, while others did not. Data availability also varied across micronutrients; for example, we often had to use proxies for Ca data, but rarely for protein. Proxies were selected based on the authors’ knowledge of local foods and an appropriate counterpart for which nutrient composition data existed; however, because we were unable to compare the nutritional composition of the local foods with their proxies, we do not know the degree of accuracy of this selection process. We often used weighted averages to fill in missing nutritional information for items in a food category when reliable local data existed. For example, we calculated weighted averages for missing wildlife species using data obtained from a laboratory analysis of the nutritional composition of Malagasy wildlife species (Supplemental Table S3).

Individual nutrient intakes were compared with each individual’s EAR. The Institute of Medicine of the National Academies defines the EAR as the average daily nutrient intake level needed to meet the requirements of half of the healthy individuals in a group. We obtained the EAR for various age and sex groups, as well as pregnant and lactating women, from the most recent Institute of Medicine tables available. We note that EAR are constructed with a focus on the median individual’s requirements, in contrast to RDA and Adequate Intakes, which are levels of nutrient intake sufficient to meet the needs of 97–98 % of all healthy individuals in a group^(^^18^^)^. We focus on EAR in the current analysis because they are appropriate for international nutritional assessment^(^^19^^)^. We note, however, that the prevalence of nutrient inadequacy may thereby be underestimated. We thus regard the inadequacy prevalences given in Table 1 as conservative lower-bound estimates. In addition, for fats we report RDA because there are no established EAR^(^^20^^)^. We utilize energy requirements for very physically active individuals^(^^21^^)^. We calculate energy intake per capita and also per adult male equivalent. Per capita energy intake was low in part because the Malagasy population is heavily skewed towards younger individuals, with the median age of the study population being less than 16 years old. Adult male equivalents are calculated by dividing an individual’s total energy requirement by the energy requirement of an adult male^(^^22^^)^.

The dietary intake data also allow for the calculation of HDDS, FCS and MDD-W. The HDDS is calculated by summing the number of food groups consumed by the household in each day, out of 12 total: (i) cereals; (ii) roots and tubers; (iii) vegetables; (iv) fruits; (v) meat, poultry and offal; (vi) eggs; (vii) fish and seafood; (viii) pulses, legumes and nuts; (ix) milk and milk products; (x) oils and fats; (xi) sugar and honey; and (xii) miscellaneous foods, including condiments, coffee, salt and spices^(^^23^^)^. HDDS questions are usually asked retrospectively of the past 24 h; our diet data set allows us to calculate daily household scores for all days in the nine-month period. FCS uses slightly different categories and applies ‘nutrient density’ weights to each (in parentheses): ‘main staples’ (2), which includes cereals, roots and tubers; pulses, legumes and nuts (3); vegetables (1); fruits (1); meat, fish and eggs (4); milk and other dairy products (4); sugar and honey (0·5); and oils and fats (0·5)^(^^24^^)^. The composite FCS is calculated by multiplying, for each group, the amount of days in the past 7 d that a household consumed a given food group by the food group’s weight and then summing these values. The FCS thus ranges from 0 to 112. Typically, scores ≤ 21 are considered ‘poor,’ scores between 21·5 and 35 are ‘borderline’ and scores > 35 are ‘acceptable’, although the universality of these thresholds has been questioned^(^^25^^)^.

We also used the estimated individual dietary intake data to calculate MDD-W for women of reproductive age. The MDD-W, which is also based on food group consumption, allocates foods into ten different categories and consuming fewer than five indicates low dietary diversity. This indicator also can reflect micronutrient adequacy across eleven micronutrients: vitamin A, thiamin, riboflavin, niacin, vitamin B_6_, folate, vitamin B_12_, vitamin C, Ca, Fe and Zn^(^^26^^)^.

## Results

### Nutrient intake

**Fig. 2:**
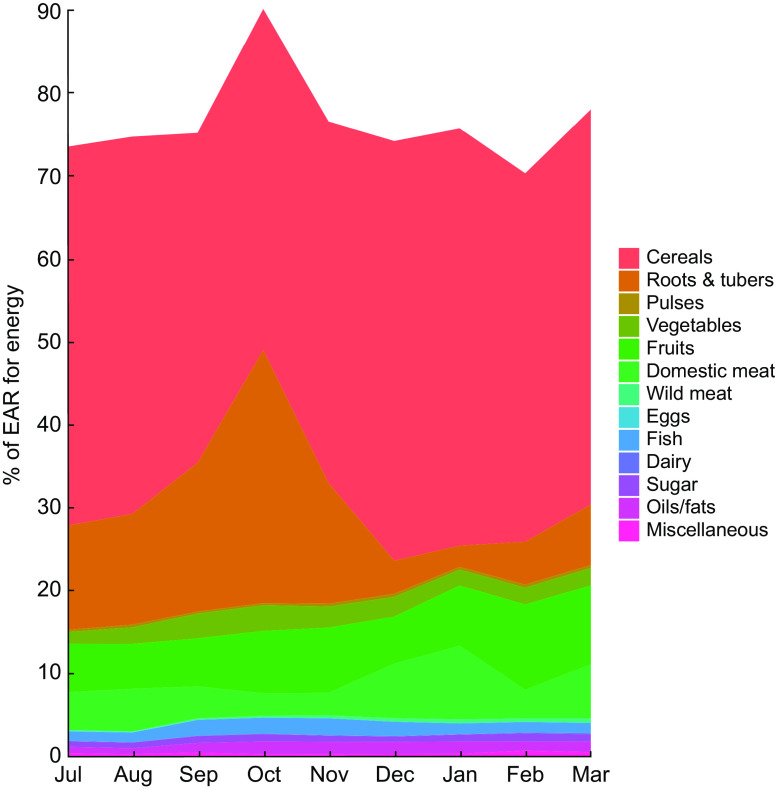
Percentage of the Estimated Average Requirement (EAR) for energy supplied by different food groups in the diet of 719 individuals of both sexes and all ages, north-eastern Madagascar, July 2013 to March 2014

We found total mass of food consumption to be relatively low, with individuals receiving a mean of only 5966 kJ (1426 kcal; 958 g) of prepared food each day, or 7770 kJ (1857 kcal; 1247 g) per adult male equivalent (Fig. 2). Rice comprised 63·1 % of all food eaten by weight and 62·2 % of total energy. Combining the weight of all cereals (rice, bread, pasta, flour) and roots and tubers (cassava, yam, taro, sweet potato, etc.), 77·3 % of all food by weight consumed in the household came from micronutrient-poor starches (Supplemental Table S4), amounting to 78·9 % of total energy. Approximately 5·3 % of all food consumed by weight was from animal-source foods, with wildlife contributing 40 % of that value. In general, the Malagasy diet in these communities is characterized by very high carbohydrate intake, sufficient protein intake, and very low fat intake.

We used nutrient composition profiles to convert daily dietary records into total nutrient intake. We found that the patterns of household food consumption varied greatly by nutrient, with some nutrients inadequately consumed, some near the sufficiency threshold and others sufficient (Table 1). On average across the year, we observed inadequate intakes for energy, vitamin A, vitamin B_12_, vitamin D, vitamin E, riboflavin, folate, Ca and Na, and sufficiency for protein, carbohydrates, vitamin C, P, niacin, Mg, Fe and Zn. Over the 5066 calculations of monthly individual intakes, the median individual could be expected to consume only 65·6 % of his/her monthly EAR for energy. Of greatest concern due to severe deficits are energy, Ca and vitamins A, B_12_ and D. Note also that median sufficiency levels are considerably lower than means for many nutrients, especially protein, vitamin A, riboflavin, vitamin B_12_ (especially) and Na. This suggests strong inequality of nutrient intake across the population. Thiamin and Na fluctuated seasonally between adequacy and inadequacy. We provide summary statistics for nutrients that do not have an EAR in Supplemental Table S5.

**Fig. 3:**
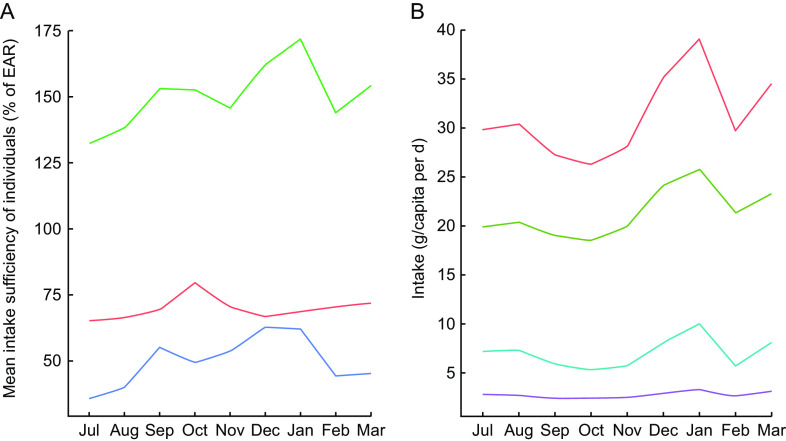
Seasonal nutrient intake patterns in the diet of 719 individuals of both sexes and all ages, north-eastern Madagascar, July 2013 to March 2014: (A) mean intake sufficiency of individuals according to the Estimated Average Requirement (EAR) for protein (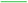), energy (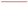) and vitamin B_1__2_ (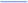); (B) per capita intake of total fat (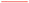), SFA (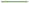), MUFA (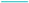) and PUFA (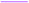)

Even with significant day-to-day variation, there were observable patterns of seasonal nutrient intake. This is attributable to the pronounced variation in rainfall, temperature, agricultural cycles and food availability. Many types of micronutrients and vitamins demonstrated such seasonality, with protein, vitamin A and vitamin B_12_ being especially volatile (Fig. 3A) and fat consumption being similarly volatile (Fig. 3B). Food consumption tends to peak during the hot season from September through January. Interestingly, for many nutrients, increased intake was directly related to increased variability – meaning that food-insecure households tended to have smoother and less variable consumption (Supplemental Fig. S1).

For the household sample as a whole, in each individual season, day-to-day nutrient intake generally approximated a random walk for all inadequately consumed nutrients, wherein a day of high consumption is likely to be followed by a day of low consumption, and vice versa. This suggests smoothing of food intake over a roughly 2–3 d period to compensate for low consumption on certain days (Supplemental Fig. S2). Regardless of variation across food groups (i.e. meat consumption is highly volatile, cereal and vegetable consumption stable with daily intake, etc.), there is a clear trend of increased nutrient intake during the hot season in Madagascar, spanning from September through January, in contrast to the colder season (Fig. 3A). Across all households, there is a clear trend of reduced intake of protein, vitamin A, vitamin B_12_, Zn and fat beginning in February and lasting until September.

**Fig. 4:**
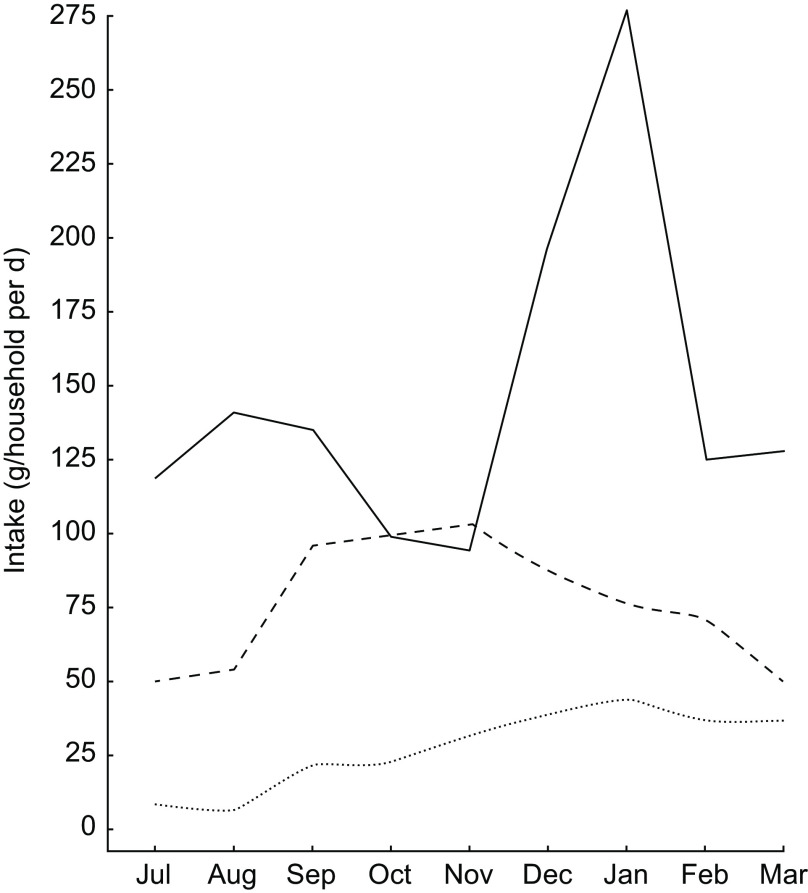
Seasonal patterns in the intake of animal-source foods (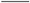, domestic meat 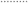 wild meat 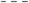, wild fish) in the diet of 719 individuals of both sexes and all ages, north-eastern Madagascar, July 2013 to March 2014

Given that these types of nutrients are found in high concentrations in animal-source foods, this trend highlights the critical importance of tenrec and lemur hunting during the periods of low consumption of domesticated meats and fish, as they are commonly hunted during this season (Fig. 4). We observed that the standard deviation of consumption across households tends to be much greater for domestic meats, as compared with fish and wild meat. Rich households tend to have access to high quantities of domestic meat, while poor households do not; all households, however, have generally the same degree of access to wild meat, thus serving a more important role in nutrient delivery in poorer households. Approximately 10·2 % of all energy comes from animal-source foods. Of animal-source energy, 28·9 % comes from wild meat and fish. Wild meats and fish also provide 16·9 % of protein, 5·8 % of Fe, 4·7 % of Zn, 5·2 % of PUFA, 16·2 % of Ca, 64·7 % of vitamin B_12_ and 71·7 % of consumed vitamin D.

Every time energy was sufficient, protein was also sufficient in the diet. Both carbohydrate and protein consumption were always sufficient throughout the year. Overall, fat consumption was very low (Fig. 3B and Supplemental Table S5), with the exception of a few holiday or ritual feasts over the course of the year, primarily sourced from rice, domesticated meats, cooking oil and avocado. Thus, a deficit in fat consumption drove the persistent inadequacy in energy intake. The recommended daily allowance for fat is between 20 and 35 % of the total energy supply, equalling 66–116 g/d for very active men and 49–86 g/d for very active women^(^^20^^)^. Mean and median daily per capita consumption are significantly below those levels (Supplemental Table S5).

### Household dietary diversity and food consumption scores

The mean HDDS over the sample period ranged between 2·4 and 6·7 (out of a possible maximum of 12), with very low variance across households. The mean and median household consumed four food groups in a day which is considered moderately diverse, while seven food groups could be seen as a low target for a healthy diverse diet. Cereals (rice) are consumed daily, while roots/tubers, on average, are consumed on 44 % of days. The least frequently consumed food group was eggs, being eaten on only 3·1 % of days, followed by dairy which was consumed on only 3·9 % of days. HDDS was also remarkably stable throughout the year, seemingly insensitive to seasonal variation (Supplemental Fig. S3).

According to the FCS thresholds, 54·4 % of household-weeks in the sample showed acceptable food consumption, 41·5 % had borderline consumption and 4·1 % of weeks had poor consumption (Supplemental Fig. S4). However, many of the weekly household experiences in the borderline category fell so close to the threshold that a slight shift in the subjective threshold would have led to a drastic increase in the prevalence of household-weeks in the ‘poor’ category. These categorical conclusions are thus somewhat arbitrary, highly sensitive to threshold choice.

A total of 166 women of reproductive age (between 15 and 49 years old) are in our sample, totalling 36 662 women-days for which consumption was reported between July 2013 and March 2014. Of these days, according to the MDD-W thresholds, only 8·4 % (*n* 3098) of women-days meet the standard of micronutrient adequacy of at least five food groups (Supplemental Fig. S5).

## Discussion

The Malagasy people living in north-eastern Madagascar face a multidimensional nutritional challenge. They have sufficient intake of some nutrients (protein, carbohydrates, vitamin C, P, niacin, Mg, Fe and Zn) and insufficient intake of others (energy, vitamin A, vitamin B_12_, vitamin D, vitamin E, riboflavin, folate, Na and Ca). The diet is characterized by sufficient protein intake (largely from rice intake), very high carbohydrates intake, and very low fat intake. Thus, there was a consistent deficit in energy throughout the year, largely attributable to inadequate fat intake linked to insufficient access to animal-source foods or other sources of fats such as cooking oil. These results are similar to what would be expected based on the FAO’s Food Balance Sheets that represent national dietary intakes^(^^27^^)^. FAO Food Balance Sheets projections were consistent with our calculations of dietary intake for Ca, riboflavin, niacin, folate, and vitamins A, C and B_12_, while they underestimated Zn and thiamin intake in comparison to our calculations^(^^27^^)^. Both methods denote severe intake inadequacies which beg for food-based interventions. This leads to important policy implications concerning the development of strategies to increase the productivity of agricultural foods and interventions to increase access to food items that could provide overall nutritional sufficiency.

It is also important to understand how households may cope with scarcity or food insecurity^(^^13^^)^. Households differ in the quantity and quality of their stocks of human, physical, natural, financial and social capital. Our results show that food-insecure households have smoother consumption; increased nutrient intake is linearly associated with increased variation in nutrient intake (Supplemental Fig. S1). Part of this is attributable to cultural factors whereby food-secure households are more likely to eat a more diverse diet and have the luxury to purchase foods from other households and adequate land to produce a variety of crops. This flexibility in diet is a phenomenon that should be examined to determine how proposed interventions may disrupt the smooth consumption patterns of food-insecure households.

Food-insecure households may also be more vulnerable to shocks and unexpected food deficits. As food-insecure households are often heavily reliant on wild foods (including wildlife), they may be disproportionately affected by changes in access to these wild foods from environmentally destructive practices and events (i.e. deforestation, unsustainable hunting, cyclones, etc.)^(^^6^^,^^7^^,^^13^^)^. In contrast, it is possible that these wild foods may be more susceptible to change from deforestation or hunting, but may be more resilient to cyclones and other forms of climate change, and thus protect food-insecure households^(^^5^^–^^7^^)^. To understand the impact of environmental changes on food systems and food security, it is necessary to have more detailed, higher-frequency data on the types of food people are consuming^(^^16^^)^. There are significant differences in the nutrient content and resilience to environmental change among foods, even those within the same broad category (i.e. shrimp *v*. wildlife; squash *v*. palm hearts), although commonly used food security indicators like the HDDS and FCS would categorize them equivalently.

There are several limitations to the present study that may lead to errors in nutrient intake estimations. Our dietary records may underestimate true consumption given the low energy intake values suggested. Given the level of agricultural labour locally, very active men would require 12 552 kJ (3000 kcal) daily, approximately 60 % more than what is currently being consumed. It is possible that our researchers did not meticulously denote when members of the household were absent for meals, often men working in the fields, leading to estimations of smaller portions of food for actually present household members. Furthermore, although validated locally to these communities, it is possible that allocating household consumption by body weight may be an inaccurate assumption. Lack of adequate nutrient composition data for many locally endemic foods was also a challenge in this research, driving us to use established proxies that may not accurately represent the nutrient content of local foods^(^^28^^)^. Additionally, because most food composition tables do not have separate values for each amino acid but rather an overall protein value^(^^4^^)^, it is possible that some amino acids are inadequate even if protein is deemed adequate. This may be problematic in the Malagasy diet where the majority of protein is consumed from rice. Given the lack of variation in protein sources, the diet may be inadequate in lysine, an amino acid found exclusively in animal-source foods and known to be important in growth and development^(^^29^^)^.

Although our consumption calculations may be somewhat underestimated, we used the EAR (median individual’s requirement) and not the RDA (nutrients adequate to satisfy 97 % of the population) to determine nutritional adequacy, and thus suggest that all of the nutrients estimated to be inadequate are of concern and some of the nutrients projected to be sufficient may actually be inadequate. The impact of the intake inadequacies observed in the dietary data likely contributes to nutritional deficiencies and a significant burden of disease. Deficits from inadequate energy intake (in this case, not enough fats) can lead to stunting, wasting, lethargy and increased risk of infection, although it is difficult to tease apart the isolated impacts of energy inadequacies from micronutrient inadequacies as it would be nearly impossible to have the former without the latter. Insufficient vitamin A intake leads to night blindness and increased mortality from infectious disease^(^^30^^)^. In addition, infants breast-fed by a vitamin B_12_-deficient mother are at risk for severe developmental abnormalities, growth failure and anaemia^(^^31^^)^. Vitamin D and Ca are both important for the development of strong bones and teeth, and for preventing long-latency diseases such as heart disease, diabetes and others^(^^32^^,^^33^^)^. Vitamin E, too, is linked to a reduced risk of CHD^(^^34^^)^. Folate deficiencies have severe consequences including congenital malformations, neural tube defects, a variety of mental disorders including Alzheimer’s disease, colorectal cancer and the development of chronic disease in later life^(^^35^^)^. Each of these deficiencies may be debilitating the health and well-being of the Malagasy people and compromising their lives and livelihoods. Also, each deficiency is preventable through food-based interventions to bolster the amounts of food and diversity in the local diet.

Interventions to stem these apparent micronutrient deficiencies could be multipronged. Vitamin A supplementation, with visits twice per year, is already successfully operating in these two communities and across the nation of Madagascar. This is important as even commonly managed nutrient deficiencies, such as iodine, continue to plague the Malagasy population^(^^36^^)^. Aside from single nutrient solutions, poultry interventions in this region have begun to prove effective, increasing the productivity of chickens and the consumption of poultry, providing necessary fats and vitamins A and B_12_^(^^7^^)^. Forms of sustainable ecosystem management, like the enforcement of regulations around tenrec hunting (the most fecund mammal on earth), could improve the productivity of the tenrec population available locally and provide a rich source of meat to local people. Additionally, increased processing of seafood products for on-site drying and smoking could allow more remote communities inland to have access to a rich source of micronutrients. Knowledge of the nutritional composition of certain types of food biodiversity could be important in dietary planning based on local availability. For instance, *Moringa olifeira* is highly nutritious^(^^37^^)^ and abundant locally.

For certain acute deficiencies that are particularly worrisome at sensitive life stages (e.g. infancy, pregnancy, etc.), health-care professionals should be aware of the dietary limits of the local food system and recommend eating particular types of food or providing at-risk patients with vitamins or supplements. Inadequate access to nutritious food is particularly worrisome given Madagascar’s top ranking among countries likely to experience civil conflict stemming from tensions between biodiversity conservation and food security^(^^38^^)^. Policy makers must be careful to create culturally sensitive development initiatives that do not compromise local conservation values or jeopardize the role that integrated wild foods and agricultural foods currently play in the diet. Pairing ecosystem-based approaches with economic development and market-based approaches to develop a food system that reinforces principles of ecosystem health and human health is of paramount importance.

## Supplementary material

For supplementary material accompanying this paper visit https://doi.org/10.1017/S1368980019001083. click here to view supplementary material
