# Supplementary material for: Seasonal trends of nutrient intake in rainforest communities of north-eastern Madagascar
Source: Public Health Nutr. 2019 Aug;22(12):2200–9. doi: 10.1017/S1368980019001083 (PMC10260550; doi:10.1017/S1368980019001083)
Supplement: Supplementary file 1 [file S1368980019001083sup001.pdf]

ONLINE SUPPLEMENTAL MATERIAL

TABLES:

Supplemental Table S1: Data sources for compiling information of nutrient content for foods in the Malagasy diet

References	Citations
US Department of Agriculture, Agricultural Research Service, Nutrient Data Laboratory. USDA National Nutrient Database for Standard Reference, Release 28.	101
Lukmanji, Z., E. Hertzmark, N. Mlingi, V. Assey, G. Ndossi, and W. Fawzi. 2008. Tanzania food composition tables. Muhimbili University of Health and Allied Sciences, Tanzania Food and Nutrition Center, Harvard School of Public Health, First Edition.	59
Korkalo, L., H. Hauta-alus, and M. Mutanen. 2011. Food composition tables for Mozambique. Department of Food and Environmental Sciences, University of Helsinki, Finland	26
Stadlmayr et al. 2010. Composition of selected foods from West Africa. Food and Agriculture Organization of the United Nations	16
Food Plant Solutions Rotarian Action Group. Potentially important food plants of Madagascar. Food Plant Solutions Field Guide - Madagascar, Version 1, September 2015	15
Food Standards Australia. 2010. Nutrient tables for use in Australia (NUTTAB)	12
Institute of Nutrition, Mahidol University (2014). ASEAN Food Composition Database, Electronic version 1, February 2014, Thailand.	10
Stadlmayr et al. 2012. West African food composition table. Food and Agriculture Organization of the United Nations, Rome.	10
Finke, M.D. 2004. Nutrient content of insects. Encyclopedia of Entomology. Scottsdale, Arizona, USA	4
Menchu, M.T., and H. Mendez. 2007. Tabla de composicion de alimentos de Centroamerica. INCAP 2nd Edition.	3
Food and Agriculture Organization of the United Nations. 1972. Food composition table for use in East Africa. US Department of Health, Education, and Welfare.	2
Hotz, C., A. Lubowa, C. Sison, M. Moursi, and C. Loechl. 2012. A food composition table for central and eastern Uganda. HarvestPlus, Washington DC.	2
Kunchit, J., P. Puwastien, A. Nitithamyong, P. Sridonpai, A. Somjai. Institute of Nutrition, Mahidol University 2015. Thai Food Composition Database, Online version 1, January 2016, Thailand.	2
Banjo, A.D., Lawal, O.A., and E. A. Songonuga. 2006. The nutritional value of fourteen species of edible insects in southwestern Nigeria. African Journal of Biotechnology 5(3): 298-301.	1
Bilgin Ş and ZUC Fidanbaş, 2011. Nutritional properties of crab (Potamon potamios Olivier, 1804) in the lake of Eğirdir (Turkey). Pak Vet J, 31(3): 239-243.	1
Edem, D.O., O.U. Eka, and E.T. Ifon. 1984. Chemical evaluation of the nutritive value of the raffia palm fruit. Food Chemistry 15(1): 9-17	1
Gobble, R., M. Taylor, and G. Lyons. 2010. Factsheet no. 10 chilli leaf. Australian Centre for International Agricultural Research.	1

The number of citations for each database used in the compilation of nutritional information for the 250 foods included in the daily household diet records. Citations include any time a database was used for nutritional information for a food, regardless of whether the source was the primary, secondary, tertiary, or quaternary source of nutritional information for that food.

Supplemental Table S2: List of food categories, and count of food items recorded in each category in the daily household diet records.

Food category	Count
**Bananas**	14
**Beans**	13
**Beverages**	2
**Bread**	9
**Bush meat**	22
**Domestic meat/poultry**	9
**Dried seafood**	5
**Dried freshwater fish**	1
**Freshwater fish**	5
**Eggs**	2
**Fruits**	30
**Greens**	33
**Insects**	6
**Nuts**	2
**Palm hearts**	28
**Pasta**	2
**Rice**	3
**Seafood**	5
**Spices**	2
**Staples**	8
**Store-bought goods**	7
**Sugar**	4
**Vegetables**	14
**Wild birds**	24
**Total**	250

Supplemental Table S3: Nutrient composition table created for northeastern Madagascar diet

Table 1a. Nutrient composition database……………………….pg. 2
Table 1b. Food categories………………………………………….....pg. 47
Table 2. Proxied food items………………………………………..pg. 48
Table 3. References…………………………………………………….pg. 56

Table 1a. Nutrient composition database

ID	Variable	Malagasy Name	English Name	Category	Proxy? (1=yes, 0=no)	Notes on proxy	Energy, kcal	Protein Content (g)	Fat (g)	Carbohydrates (g)
**1**	fonmant	Fontsy manta	Banana (plantain), boiled	Bananas	0		89.00	1.10	0.30	22.80
**2**	lavrav	Lava ravina	Banana (plantain), boiled	Bananas	0		156.00	1.20	0.30	31.20
**3**	fonamb	Fontsy Ambarababoaka	Latundan Banana	Bananas	1	Traditional banana cooked	116.00	0.80	0.20	22.80
**4**	fonand	Fontsy andatra	Latundan Banana	Bananas	1	Traditional banana cooked	116.00	0.80	0.20	22.80
**5**	fonbit	Fontsy bitavia	Traditional banana	Bananas	1	Traditional banana cooked	116.00	0.80	0.20	22.80
**6**	fonkir	Fontsy kiriva	Banana (plantain) cooked	Bananas	1	Plantain, ripe, boiled	156.00	1.20	0.30	31.20
**7**	fonla	Fontsy lahy	Banana (plantain) cooked	Bananas	1	Plantain, ripe, boiled	156.00	1.20	0.30	31.20
**8**	fonmak	Fontsy makoa	Red Banana	Bananas	1	Traditional banana cooked	116.00	0.80	0.20	22.80
**9**	fonrav	Fontsy malamaravina	Latundan Banana	Bananas	1	Traditional banana cooked	116.00	0.80	0.20	22.80
**10**	fontsam	Fontsy tsameroa	Banana (plantain)	Bananas	1	Plantain, ripe, boiled	156.00	1.20	0.30	31.20
**11**	fontsami	Fontsy tsaminambona	Banana (plantain)	Bananas	1	Plantain, ripe, boiled	156.00	1.20	0.30	31.20

	Variable	Malagasy Name	English Name	Category	Proxy? (1=yes, 0=no)	Notes on proxy	Energy, kcal	Protein Content (g)	Fat (g)	Carbohydrates (g)
**12**	fontsiam	Fontsy tsiambotsihiva	Banana (plantain)	Bananas	1	Plantain, ripe, boiled	156.00	1.20	0.30	31.20
**13**	fontsil	Fontsy lilaka	Banana (plantain)	Bananas	1	Plantain, ripe, boiled	156.00	1.20	0.30	31.20
**14**	varfon	Vary sy fontsy	Rice and banana mixture	Bananas	1	Assumed even weight of traditional banana and rice and averaged the nutritional composition of those two.	125.50	1.90	0.50	29.80
**15**	har	Haricot	Kidney beans	Beans	0		127.00	8.70	0.50	22.80
**16**	lojo	Lojo	Cowpeas, boiled	Beans	0		137.00	8.60	0.50	22.30
**17**	pista	Pistaches	Peanuts	Beans	0		333.00	15.00	22.01	21.26
**18**	voabor	Voanjobory	Chickpeas	Beans	0		164.00	8.90	2.60	27.40
**19**	antsot	Antsotry	Type of beans	Beans	1	Green bean, boiled without salt	166.00	11.40	0.70	19.70
**20**	antvong	Antidahivondraka	Type of beans	Beans	1	Weighted average: cowpeas, kidney, mung, lima beans	119.00	7.81	0.42	20.78
**21**	bethaz	Betanihazo	Type of beans	Beans	1	Cowpeas, boiled without salt	96.99	3.20	0.50	22.30
**22**	grop	Gros pois	Type of beans	Beans	1	Lima beans	115.00	7.80	0.38	20.88

	Variable	Malagasy Name	English Name	Category	Proxy? (1=yes, 0=no)	Notes on proxy	Energy, kcal	Protein Content (g)	Fat (g)	Carbohydrates (g)
**23**	telvo	Telovolana	Type of beans	Beans	1	Cowpeas, boiled without salt	96.99	3.20	0.50	22.30
**24**	tsias	Tsiasisa	Type of beans	Beans	1	Mung beans, boiled, without salt	105.00	7.02	0.38	19.15
**25**	vam	Vamaho	Black sesames	Beans	1	Sesame seed, unspecified white or black	573.00	17.70	49.70	23.50
**26**	voamai	Voamaina	Beans (not specified)	Beans	1	Kidney beans (mature, boiled, without salt)	127.00	8.70	0.50	22.80
**27**	voatsi	Voantsiroko	Type of beans	Beans	1	Cowpeas, boiled without salt	96.99	3.20	0.50	22.30
**28**	cafe	Café	Coffee, with milk, without sugar	Beverages	0		11.50	1.30	1.60	0.20
**29**	ronon	Ronono	Milk, cow, whole, fresh	Beverages	0		60.00	3.20	3.30	4.50
**30**	mofvar	Mofo vary	Rice bread	Bread	0		239.10	2.20	7.50	41.10
**31**	beig	Beignets	Doughnuts	Bread	1	Wheat, dough, deep fried	317.00	7.20	7.70	53.40
**32**	belar	Belaro	Type of bread	Bread	1	English muffin	223.00	8.70	2.00	44.80
**33**	mofbok	Mofo bokety	Type of bread	Bread	1	Cassava flour	274.00	8.80	3.00	51.90
**34**	mofgas	Mofo gasy	Type of bread	Bread	1	Wheat, dough, deep fried	317.00	7.20	7.70	53.40
**35**	mofo	Mofo	Bread	Bread	1	Sourdough bread	273.00	8.60	6.30	46.00

	Variable	Malagasy Name	English Name	Category	Proxy? (1=yes, 0=no)	Notes on proxy	Energy, kcal	Protein Content (g)	Fat (g)	Carbohydrates (g)
**36**	mofon	Mofo fontsy	Bread	Bread	1	Sourdough bread	273.00	8.60	6.30	46.00
**37**	mofrav	Mofo ravina	Rice and banana bread	Bread	1	Assumed even weight of traditional banana and rice and averaged the nutritional composition of those two.	125.50	1.90	0.50	29.80
**38**	mofvzah	Mofo vazaha	Flour bread	Bread	1	Sourdough bread	273.00	8.60	6.30	46.00
**39**	bokom	Bokombolo	Eastern lesser bamboo lemur	Bushmeat	0		138.30	23.1	4.38	0.00
**40**	sokin	Sokina	Greater hedgehog tenrec	Bushmeat	0		112.80	14.42	4.38	0.00
**41**	trandr	Trandraka	Tailless tenrec	Bushmeat	0		107.18	15	4.38	0.00
**42**	varkos	Gidro	White-headed lemur	Bushmeat	0		105.20	15.6	4.38	0.00
**43**	ampon	Fotsife	Eastern woolly lemur	Bushmeat	1	Hapalemur griseus	138.30	23.1	4.38	0.00
**44**	andreh	Andrehy	Food Plant Solutions Rotarian Action Group 2008agascan rousette	Bushmeat	1	Pteropus rodricensis	216.43	26.475	4.38	0.00
**45**	antsaora	Antsaora	Lowland streaked tenrec	Bushmeat	1	Setifer setosus	112.80	14.42	4.38	0.00
**46**	ayeaye	Aye-aye	Aye-aye	Bushmeat	1	Eulemur albifrons	105.20	15.6	4.38	0.00

	Variable	Malagasy Name	English Name	Category	Proxy? (1=yes, 0=no)	Notes on proxy	Energy, kcal	Protein Content (g)	Fat (g)	Carbohydrates (g)
**47**	babkot	Babakoto	Indri, babakoto, primate	Bushmeat	1	Hapalemur griseus	138.30	23.1	4.38	0.00
**48**	bib	Bibidia	Wildlife (unspecified)	Bushmeat	1	Eulemur albifrons	105.20	15.6	4.38	0.00
**49**	falanok	Falanoka	Falanouc, rare mongoose-like mammal, endemic	Bushmeat	1	Weighted average of all bushmeat	135.98	18.919	4.38	0.00
**50**	fosa	Fosa	Cat-like, carnivous mammal, endemic	Bushmeat	1	Weighted average of all bushmeat	135.98	18.919	4.38	0.00
**51**	jaboa	Jaboady	Small Indian civet	Bushmeat	1	Weighted average of all bushmeat	135.98	18.919	4.38	0.00
**52**	kary	Kary	Wild cat	Bushmeat	1	Weighted average of all bushmeat	135.98	18.919	4.38	0.00
**53**	lambodia	Lambo dia	Bushpig	Bushmeat	1	Game meat, boar, wild, cooked, roasted	160.00	28.30	4.38	0.00
**54**	radak	Radaka	Leopard frog	Bushmeat	1	Frog legs, raw	73.00	16.00	0.30	0.00
**55**	tomb	Tombokontsodiny	Malagasy civet	Bushmeat	1	Weighted average of all bushmeat	135.98	18.919	4.38	0.00
**56**	tongo	Tongo	Red-bellied lemur	Bushmeat	1	Eulemur albifrons	105.20	15.6	4.38	0.00
**57**	tsid	Tsidy	Brown mouse lemur	Bushmeat	1	Eulemur albifrons	105.20	15.6	4.38	0.00
**58**	tsits	Tsitsiha	Greater dwarf lemur	Bushmeat	1	Eulemur albifrons	105.20	15.6	4.38	0.00

	Variable	Malagasy Name	English Name	Category	Proxy? (1=yes, 0=no)	Notes on proxy	Energy, kcal	Protein Content (g)	Fat (g)	Carbohydrates (g)
**59**	varkan	Varikandavaka	Lepilemur sp.	Bushmeat	1	Hapalemur griseus	138.30	23.1	4.38	0.00
**60**	vontsi	Vontsira	Ring-tailed mongoose	Bushmeat	1	Weighted average of all bushmeat	135.98	18.919	4.38	0.00
**61**	bol	Bolety	Ground beef	Domestic meat/poultry; pork/beef	0		212.00	27.80	11.20	0.00
**62**	henam	Henandambo	Pork	Domestic meat/poultry; pork/beef	0		550.00	28.90	52.70	0.00
**63**	henom	Henanomby	Beef	Domestic meat/poultry; pork/beef	0		209.00	36.20	7.20	0.00
**64**	angozy	Angozy	Zebu skin and fat (indicine cattle, humped cattle, Brahman)	Domestic meat/poultry; pork/beef	1	Beef, subcutaneous fat, cooked	731.00	6.50	78.30	0.00
**65**	draki	Drakidraky	Duck, domesticated, meat and skin, cooked, roasted	Domestic meat/poultry; poultry	0		337.00	18.99	28.35	0.00
**66**	kolok	Kolokoloko	Turkey	Domestic meat/poultry; poultry	0		189.00	28.55	7.39	0.06
**67**	sadok	Sadoko	Duck	Domestic meat/poultry; poultry	0		337.00	18.99	28.35	0.00
**68**	voran	Vorondrano	Duck	Domestic meat/poultry; poultry	0		337.00	18.99	28.35	0.00
**69**	ako	Akoho	Chicken	Domestic meat/poultry	1	Chicken, dark meat	268.00	22.00	20.00	0.00

	Variable	Malagasy Name	English Name	Category	Proxy? (1=yes, 0=no)	Notes on proxy	Energy, kcal	Protein Content (g)	Fat (g)	Carbohydrates (g)
**70**	lkmama	Laoko ranomamy maina	Freshwater fish	Freshwater fish	0		335.00	58.60	9.40	0.00
**71**	ormain	Orana maina	Dried shrimp	Dried seafood	0		301.00	55.80	2.40	13.10
**72**	ankor	Anankorana	Dried white shrimp	Dried seafood	1	(Palaemon spp; Penaeus sp.)	316.00	69.10	4.00	1.00
**73**	lkmasma	Laoko ranomasina maina	Dried saltwater fish	Dried seafood	1	Fish, dried, cod	368.00	79.80	3.20	0.00
**74**	patsa	Patsa	Dried red shrimp	Dried seafood	1	(Palaemon spp; Penaeus sp.)	316.00	69.10	4.00	1.00
**75**	lksir	Laoko sira	Salted fish (typically larger fish)	Dried seafood	1	Dried saltwater fish	368.00	79.80	3.20	0.00
**76**	atako	Atody akoho	Chicken egg, fried	Eggs	0		245.00	11.80	21.60	1.00
**77**	atovor	Atody vorona	Duck eggs	Eggs	0		185.00	12.81	13.77	1.45
**78**	lkmaml	Laoko ranomamy legny	Freshwater fish	Freshwater fish	0		84.00	14.80	2.30	0.00
**79**	amal	Amalona	Eel	Freshwater fish	1	Anguilla spp. (river eels), cooked, 50% fat/50% lean	244.50	15.00	20.00	0.00
**80**	anvil	Anvil	Freshwater fish	Freshwater fish	1	Small, freshwater fish	84.00	14.80	2.30	0.00
**81**	foza	Foza	Crab, freshwater	Freshwater fish	1	Potamon potamios (lake crab)	85.00	13.94	0.67	0.60
**82**	sambern	Sambireny	Freshwater fish	Freshwater fish	1	Fish, small, freshwater	84.00	14.80	2.30	0.00
**83**	akoh	Akohorano	Seasoning	Fruits	0		0.00	0.00	0.00	0.00

	Variable	Malagasy Name	English Name	Category	Proxy? (1=yes, 0=no)	Notes on proxy	Energy, kcal	Protein Content (g)	Fat (g)	Carbohydrates (g)
**84**	ampal	Ampalibe	Jackfruit, raw	Fruits	0		94.00	1.50	0.30	24.00
**85**	ampil	Ampilimosy	Grapefruit	Fruits	0		33.00	0.70	0.10	8.40
**86**	coros	Corossol	Soursop, raw	Fruits	0		66.00	1.00	0.30	16.80
**87**	gavb	Gavobe	Guava	Fruits	0		56.86	1.10	0.40	15.30
**88**	letch	Letchi	Lychee	Fruits	0		78.00	0.80	0.40	16.90
**89**	makob	Makoba	Asian apple	Fruits	0		45.00	0.60	0.20	10.10
**90**	manas	Manasy	Pineapple	Fruits	0		48.00	0.50	0.10	12.60
**91**	manga	Manga	Mango	Fruits	0		65.00	0.50	0.30	17.00
**92**	pm	Paoma	Apple	Fruits	0		52.00	0.20	0.20	13.80
**93**	samab	Samabia	Type of fruit	Fruits	0		22.00	1.00	0.40	4.50
**94**	vapmas	Vapaza masaka	Ripened papaya	Fruits	0		39.00	0.60	0.10	9.80
**95**	voahg	Raonjy	Orange	Fruits	0		47.00	0.90	0.10	11.80
**96**	voaraf	Voandrafia	Raffia tree fruit	Fruits	0		380.00	6.10	6.12	61.40
**97**	zano	Zano	Custard apple	Fruits	0		78.60	1.70	0.60	15.80
**98**	zavok	Zavoka	Avocado	Fruits	0		160.00	2.00	14.70	8.50
**99**	mangvz	Mangambazaha	Type of fruit	Fruits	1	Mango	65.00	0.50	0.30	17.00
**100**	matbar	Matohabaratra	Type of fruit	Fruits	1	Weighted average of all fruits	91.94	1.20	6.12	10.14
**101**	sakoan	Sakoana	Plum of Cythera, apple kythira, tree Kythera	Fruits	1	Weighted average of all fruits	91.94	1.20	6.12	10.14
**102**	tomat	Tomate	Tomato, ripe	Fruits	1	Fresh, red, roma tomato	18.00	0.88	0.20	3.89
**103**	tsamkob	Tsaminakoba	Type of fruit	Fruits	1	Weighted average of all fruits	91.94	1.20	6.12	10.14
**104**	vanok	Vanonoka	Type of fruit	Fruits	1	Weighted average of all fruits	91.94	1.20	6.12	10.14

	Variable	Malagasy Name	English Name	Category	Proxy? (1=yes, 0=no)	Notes on proxy	Energy, kcal	Protein Content (g)	Fat (g)	Carbohydrates (g)
**105**	vaor	Vaory	Type of fruit	Fruits	1	Weighted average of all fruits	91.94	1.20	6.12	10.14
**106**	voafk	Voafoko	Type of fruit	Fruits	1	Weighted average of all fruits	91.94	1.20	6.12	10.14
**107**	voahgd	Voahangy Dy	Wild Orange	Fruits	1	Orange	47.00	0.90	0.10	11.80
**108**	voakz	Voankazo	Fruit (not specified)	Fruits	1	Weighted average of all fruits	91.94	1.20	6.12	10.14
**109**	voasir	Voasirindrina	Voasirindrina	Fruits	1	Weighted average of all fruits	91.94	1.20	6.12	10.14
**110**	vong	Vongo	Type of fruit	Fruits	1	Weighted average of all fruits	91.94	1.20	6.12	10.14
**111**	vontsik	Vontsikobila	Type of fruit	Fruits	1	Weighted average of all fruits	91.94	1.20	6.12	10.14
**112**	Vapman	Vapaza manta	Green papaya	Fruits	0		39.00	0.60	0.10	9.80
**113**	anamb	Ananambo	Moringa Leaves, drumstick leaves	Greens	0		60.00	5.27	0.93	11.15
**114**	anlad	Anantsalady	Watercress, raw	Greens	0		26.29	2.90	0.40	0.80
**115**	anmang	Anantsomanga	Sweet potato leaves	Greens	0		40.13	3.90	0.30	6.40
**116**	anmari	Ananamarigozy	Bitter melon, bitter gourd, bitter squash, balsam-pear, raw	Greens	0		30.00	5.30	0.69	3.29

	Variable	Malagasy Name	English Name	Category	Proxy? (1=yes, 0=no)	Notes on proxy	Energy, kcal	Protein Content (g)	Fat (g)	Carbohydrates (g)
**117**	anmen	Anamena	Red Amaranth leaves, raw	Greens	0		33.00	2.46	0.33	4.02
**118**	anreb	Anantsirebika	Squash leaves	Greens	0		19.00	3.15	0.40	2.33
**119**	ansakay	Anantsakay	Leaves of pili pili peppers	Greens	0		40.00	2.00	0.20	9.00
**120**	antsaho	Anantsahono	Taro leaves, cooked	Greens	0		22.00	5.00	0.70	6.70
**121**	anvap	Anana vapaza	Papaya leaves	Greens	0		90.34	8.00	0.40	4.90
**122**	belap	Belapotrika	Amaranth, leaves, raw	Greens	0		23.00	4.50	0.50	8.50
**123**	ravtsos	Ravina tsosoty	Chayote leaves	Greens	0		60.00	4.00	0.40	4.70
**124**	ravzah	Ravinambazaha	Cassava leaves	Greens	0		27.95	3.00	0.20	7.30
**125**	anab	Anambe	Type of greens	Greens	1	Bok Choy, raw	13.00	1.50	0.20	2.18
**126**	anan	Anana	General term for greens	Greens	1	Weighted average of all greens	36.98	3.37	0.38	6.51
**127**	anbaml	Anamabala	Type of greens	Greens	1	Weighted average of all greens	36.98	3.37	0.38	6.51
**128**	anbon	Ananabontro	Type of greens	Greens	1	Weighted average of all greens	36.98	3.37	0.38	6.51
**129**	angid	Anangidy	Type of greens	Greens	1	Weighted average of all greens	36.98	3.37	0.38	6.51
**130**	angis	Anangisa	Type of greens	Greens	1	Weighted average of all greens	36.98	3.37	0.38	6.51
**131**	anjf	Ananjofo	Type of greens	Greens	1	Weighted average of all greens	36.98	3.37	0.38	6.51

	Variable	Malagasy Name	English Name	Category	Proxy? (1=yes, 0=no)	Notes on proxy	Energy, kcal	Protein Content (g)	Fat (g)	Carbohydrates (g)
**132**	anlao	Anamafana	Toothache plant, jambu, spot plant	Greens	1	Weighted average of all greens	36.98	3.37	0.38	6.51
**133**	anpats	Anampatsy	Type of greens	Greens	1	Weighted average of all greens	36.98	3.37	0.38	6.51
**134**	antarik	Anantarika	Type of greens	Greens	1	Weighted average of all greens	36.98	3.37	0.38	6.51
**135**	antsal	Anantsalepo	Type of greens	Greens	1	Weighted average of all greens	36.98	3.37	0.38	6.51
**136**	antsek	Anantsenko	Wild ferns	Greens	1	Fiddlehead ferns	34.00	4.55	0.40	0.54
**137**	antsid	Anantsindra	White nightshade	Greens	1	Nightshade, Ethiopian (Solanum aethiopicum), fruit	32.00	1.50	0.10	7.20
**138**	antsinah	Anantsinahy	Type of greens	Greens	1	Weighted average of all greens	36.98	3.37	0.38	6.51
**139**	antsir	Anantsiriry	Rice paddy clovers	Greens	1	Weighted average of all greens	36.98	3.37	0.38	6.51
**140**	antsom	Anantsonga	Type of greens	Greens	1	Weighted average of all greens	36.98	3.37	0.38	6.51
**141**	fela	Felagna	Flowers from Hesikesika	Greens	1	Weighted average of all greens	36.98	3.37	0.38	6.51
**142**	pets	Petsay	Turnip greens	Greens	1	Brassica rapa: Turnip greens, raw	32.00	1.50	0.30	7.13

	Variable	Malagasy Name	English Name	Category	Proxy? (1=yes, 0=no)	Notes on proxy	Energy, kcal	Protein Content (g)	Fat (g)	Carbohydrates (g)
**143**	ravhets	Ravina hetsika	Type of greens	Greens	1	Weighted average of all greens	36.98	3.37	0.38	6.51
**144**	raving	Ravina angivy	Type of greens	Greens	1	Weighted average of all greens	36.98	3.37	0.38	6.51
**145**	solof	Solofoko	Type of greens (like hesikesika but wild forest version)	Greens	1	Weighted average of all greens	36.98	3.37	0.38	6.51
**146**	voant	Voantandroka	Rhinoceros beetle	Insects	0	Rhinoceros beetle adult	192.00	27.10	3.70	11.20
**147**	ankantel	Ankaninantely	Fresh honeycomb	Insects	1	Honey, raw	304.00	0.30	0.00	82.30
**148**	lafa	Lafa	Beetle larvae	Insects	1	Rhinoceros beetle adult	192.00	27.10	3.70	11.20
**149**	lafbit	Lafa bitay	Rhinoceros beetle grub	Insects	1	Rhinoceros beetle adult	192.00	27.10	3.70	11.20
**150**	lafoh	Lafa fohy	Beetle larvae	Insects	1	Rhinoceros beetle adult	192.00	27.10	3.70	11.20
**151**	tsibo	Tsibobona	No common name	Insects	1	Cybister sp.	192.00	27.10	3.70	11.20
**152**	voan	Voanio	Coconut	Nuts	0		70.00	0.50	3.40	10.00
**153**	vakor	Vakoromanga	Type of nut	Nuts	1	Peanuts, all types, boiled, with salt	318.00	13.50	22.01	21.26
**154**	ambavaka	Hovotra	Palm hearts (unspecified type)	Palm hearts	0		115.00	2.70	0.20	25.61
**155**	hov	Hovotra	Palm hearts	Palm hearts	0		115.00	2.70	0.20	25.61
**156**	hovakom	Hovotra akombambo	Palm hearts	Palm hearts	0		115.00	2.70	0.20	25.61
**157**	hovala	Hovotra alanana	Palm hearts	Palm hearts	0		115.00	2.70	0.20	25.61
**158**	hovanvi	Hovotra anivogno	Palm hearts	Palm hearts	0		115.00	2.70	0.20	25.61

	Variable	Malagasy Name	English Name	Category	Proxy? (1=yes, 0=no)	Notes on proxy	Energy, kcal	Protein Content (g)	Fat (g)	Carbohydrates (g)
**159**	hovaram	Hovotra ramironkona	Palm hearts	Palm hearts	0		115.00	2.70	0.20	25.61
**160**	hovbak	Hovotra bakoko	Palm hearts	Palm hearts	0		115.00	2.70	0.20	25.61
**161**	hovbor	Hovotra boresy	Palm hearts	Palm hearts	0		115.00	2.70	0.20	25.61
**162**	hovdana	Hovotra hadanadana	Palm hearts	Palm hearts	0		115.00	2.70	0.20	25.61
**163**	hovfan	Hovotra	Palm hearts	Palm hearts	0		115.00	2.70	0.20	25.61
**164**	hovfnmar	Hovotra fontsy maroa	Palm hearts	Palm hearts	0		115.00	2.70	0.20	25.61
**165**	hovfonlah	Hovotra fontsy lahy	Palm hearts	Palm hearts	0		115.00	2.70	0.20	25.61
**166**	hovhonk	Hovotra honkona	Palm hearts	Palm hearts	0		115.00	2.70	0.20	25.61
**167**	hovhsn	Hovotra hasina	Palm hearts	Palm hearts	0		115.00	2.70	0.20	25.61
**168**	hovjav	Hovotra javavy	Palm hearts	Palm hearts	0		115.00	2.70	0.20	25.61
**169**	hovlav	Hovotra lavavozona	Palm hearts	Palm hearts	0		115.00	2.70	0.20	25.61
**170**	hovlavm	Hovotra lavamboko	Palm hearts	Palm hearts	0		115.00	2.70	0.20	25.61
**171**	hovmaf	Hovotra mafaiky hely	Palm hearts	Palm hearts	0		115.00	2.70	0.20	25.61
**172**	hovmam	Hovotra mamy	Palm hearts	Palm hearts	0		115.00	2.70	0.20	25.61
**173**	hovmanra	Hovotra manarana	Palm hearts	Palm hearts	0		115.00	2.70	0.20	25.61
**174**	hovpalm	Hovotra palmier	Palm hearts	Palm hearts	0		115.00	2.70	0.20	25.61
**175**	hovraf	Hovotra rafia	Palm hearts	Palm hearts	0		115.00	2.70	0.20	25.61
**176**	hovrav	Hovotra ravina	Palm hearts	Palm hearts	0		115.00	2.70	0.20	25.61
**177**	hovsaror	Hovotra saroro	Palm hearts	Palm hearts	0		115.00	2.70	0.20	25.61
**178**	hovtsar	Hovotra tsaravoasira	Palm hearts	Palm hearts	0		115.00	2.70	0.20	25.61
**179**	hovtsik	Hovotra tsikorevana	Palm hearts	Palm hearts	0		115.00	2.70	0.20	25.61
**180**	hovvan	Hovotra vanio	Palm hearts	Palm hearts	0		115.00	2.70	0.20	25.61

	Variable	Malagasy Name	English Name	Category	Proxy? (1=yes, 0=no)	Notes on proxy	Energy, kcal	Protein Content (g)	Fat (g)	Carbohydrates (g)
**181**	hovvon	Hovotra vontro	Palm hearts	Palm hearts	0		115.00	2.70	0.20	25.61
**182**	longf	Longo fy	Type of pasta	Pasta	1	Pasta, homemade, with egg, cooked	130.00	5.28	1.74	23.54
**183**	pate	Pate	Pasta	Pasta	1	Pasta, homemade, with egg, cooked	130.00	5.28	1.74	23.54
**184**	varh	Vary horaka	Paddy rice	Rice	1	Brown rice, boiled	135.00	3.00	0.80	28.40
**185**	varj	Vary jinja	Swidden rice	Rice	1	Brown rice, boiled	135.00	3.00	0.80	28.40
**186**	vary_mas	Vary masaka	Cooked rice	Rice	1	Brown rice, boiled	135.00	3.00	0.80	28.40
**187**	lkmasl	Laoko ranomasina legny	Saltwater fish	Seafood	0		98.00	18.10	2.40	0.00
**188**	ordv	Orandava	Prawns	Seafood	0		108.00	17.90	3.70	0.80
**189**	orita	Orita	Octopus	Seafood	0		164.00	29.82	2.08	4.40
**190**	orlen	Orana legny	Fresh shrimp	Seafood	0		99.00	23.98	0.28	0.20
**191**	sardin	Sardines	Sardines	Seafood	0		112.00	21.40	2.30	0.00
**192**	sanktan	Sakay tany	Ginger	Spices	0		66.00	2.10	0.30	15.30
**193**	tonglay	Tongolay	Garlic	Spices	0		149.00	6.40	0.50	33.10
**194**	ambzh	Ambazaha	Cassava, dried	Staples	0		314.00	2.60	0.70	76.60
**195**	ovybe	Ovy be	Yam, boiled, without salt	Staples	0		134.00	2.00	0.20	28.90
**196**	sahogn	Sahogno	Taro, raw	Staples	0		294.10	1.96	0.20	26.50
**197**	sahomand	Sahogno mandady	Taro	Staples	0		142.00	0.50	0.10	34.60
**198**	soanb	Soanambo	Breadfruit, raw	Staples	0		103.00	1.10	0.20	27.10
**199**	tsmg	Tsomanga	Sweet potatoes	Staples	0		86.72	1.10	0.10	24.30

	Variable	Malagasy Name	English Name	Category	Proxy? (1=yes, 0=no)	Notes on proxy	Energy, kcal	Protein Content (g)	Fat (g)	Carbohydrates (g)
**200**	hanbrk	Hanimbiroka	Unspecified type of root vegetable	Staples	1	Weighted average: cassava, yam, sweet potato, taro	139.33	1.80	0.23	32.59
**201**	ovdi	Ovy dia	Wild yams	Staples	1	Yam, boiled, without salt	85.29	2.70	0.20	28.90
**202**	farine	Farine	Flour	Store-bought goods	0		358.00	10.30	1.50	73.10
**203**	jumbo	Jumbo	Jumbo bouillon chicken stock cubes	Store-bought goods	0		170.00	17.30	4.00	16.10
**204**	manka	Mankafy	Seasoning	Store-bought goods	0		0.00	0.00	0.00	0.00
**205**	menk	Menaka	Palm oil	Store-bought goods	0		862.00	0.00	100.00	0.00
**206**	sostom	Sosy tomaty	Tomato paste	Store-bought goods	0		63.00	0.00	0.00	0.90
**207**	vingr	Vinaigre	Vinegar	Store-bought goods	0		19.00	0.00	0.00	0.30
**208**	siramam	Siramamy	Sugar	Store-bought goods; sugar	0		387.00	0.00	0.00	99.90
**209**	antel	Antely	Honey	Sugar	0		304.00	0.30	0.00	82.40
**210**	fary	Fary	Sugarcane	Sugar	0		26.00	0.00	0.00	6.80
**211**	ranpar	Ranopary	Sugarcane juice	Sugar	0		56.00	0.00	0.00	14.60
**212**	rononb	Ronono boite	Condensed milk, canned	Sugar	0		566.00	6.80	7.60	10.00
**213**	tongl	Tongolo	Onions	Vegetable	0		40.00	1.30	0.20	9.20
**214**	agiv	Angivy	African eggplant, type of nightshade	Vegetable	1	Nightshade, Ethiopian (Solanum aethiopicum), fruit	32.00	1.50	0.10	7.20

	Variable	Malagasy Name	English Name	Category	Proxy? (1=yes, 0=no)	Notes on proxy	Energy, kcal	Protein Content (g)	Fat (g)	Carbohydrates (g)
**215**	cucum	Cucumber	Cucumber, with peel, raw	Vegetables	0		15.00	0.70	0.10	3.60
**216**	karaot	Karaoty	Carrots	Vegetables	0		41.00	0.90	0.20	9.60
**217**	margz	Marigozy	Bitter melon, bitter gourd, bitter squash, balsam-pear	Vegetables	0		17.00	1.00	0.17	3.70
**218**	olatr	Olatra	Mushrooms	Vegetables	0		27.00	2.20	0.50	5.10
**219**	papang	Papangay	Loofa gourd, sponge gourd	Vegetables	0		45.00	4.10	0.20	4.00
**220**	patsol	Patrola	Loofa gourd, sponge gourd	Vegetables	0		45.00	4.10	0.20	4.00
**221**	sosot	Sosoty	Chayote, boiled	Vegetables	0		24.00	0.62	0.48	5.09
**222**	tsakolen	Tsakotsako legny	Fresh corn	Vegetables	0		96.00	3.46	1.50	20.98
**223**	tsireb	Daboira	Squash	Vegetables	0		20.00	0.90	0.30	4.30
**224**	vajb	Vajabo	Watermelon	Vegetables	0		21.98	0.40	0.20	7.60
**225**	ambarbanj	Ambarabanjina	Type of vegetable in cucumber family	Vegetables	1	Cucumber, with peel, raw	15.00	0.70	0.10	3.60
**226**	voangar			Vegetables	1	Chayote, boiled without salt	24.00	0.62	0.48	5.09
**227**	fine	Finengo	Dove	Wild birds	0		213.00	23.90	13.00	0.00
**228**	akanga	Akanga	Guinea Fowl	Wild birds	1	Guinea hen, meat and skin	158.00	23.40	6.45	0.00
**229**	ambos	Ambosanga	Type of wild bird	Wild birds	1	Weighted average of all wild birds	225.20	21.52	15.08	0.00
**230**	boeza	Boeza	Lesser vasa parrot	Wild birds	1	Weighted average of all wild birds	225.20	21.52	15.08	0.00

	Variable	Malagasy Name	English Name	Category	Proxy? (1=yes, 0=no)	Notes on proxy	Energy, kcal	Protein Content (g)	Fat (g)	Carbohydrates (g)
**231**	dom	Domohina	Type of wild bird	Wild birds	1	Weighted average of all wild birds	225.20	21.52	15.08	0.00
**232**	fangad	Fangadiovy	Type of wild bird	Wild birds	1	Weighted average of all wild birds	225.20	21.52	15.08	0.00
**233**	firas	Firasa	Hawk	Wild birds	1	Weighted average of all wild birds	225.20	21.52	15.08	0.00
**234**	fod	Fody	Type of wild bird	Wild birds	1	Weighted average of all wild birds	225.20	21.52	15.08	0.00
**235**	hitsik	Hetsiketsika	Hawk	Wild birds	1	Weighted average of all wild birds	225.20	21.52	15.08	0.00
**236**	kaikmav	Kaikimavo	Type of wild bird	Wild birds	1	Weighted average of all wild birds	225.20	21.52	15.08	0.00
**237**	kilan	Kilandy	Type of wild bird	Wild birds	1	Weighted average of all wild birds	225.20	21.52	15.08	0.00
**238**	koa	Koa	Type of wild bird	Wild birds	1	Weighted average of all wild birds	225.20	21.52	15.08	0.00
**239**	lampir	Lampirana	Type of wild bird	Wild birds	1	Weighted average of all wild birds	225.20	21.52	15.08	0.00
**240**	mariah	Mariha	Type of wild bird	Wild birds	1	Weighted average of all wild birds	225.20	21.52	15.08	0.00
**241**	papa	Papango	Hawk	Wild birds	1	Weighted average of all wild birds	225.20	21.52	15.08	0.00

	Variable	Malagasy Name	English Name	Category	Proxy? (1=yes, 0=no)	Notes on proxy	Energy, kcal	Protein Content (g)	Fat (g)	Carbohydrates (g)
**242**	sob	Sobery	Type of wild bird	Wild birds	1	Weighted average of all wild birds	225.20	21.52	15.08	0.00
**243**	totor	Totoroka	Type of owl	Wild birds	1	Weighted average of all wild birds	225.20	21.52	15.08	0.00
**244**	triala	Tritrikiala	Type of wild bird	Wild birds	1	Weighted average of all wild birds	225.20	21.52	15.08	0.00
**245**	tsik	Tsikoza	Type of wild bird	Wild birds	1	Weighted average of all wild birds	225.20	21.52	15.08	0.00
**246**	tsir	Tsiriry	Type of wild bird	Wild birds	1	Weighted average of all wild birds	225.20	21.52	15.08	0.00
**247**	vorad	Vorona dy	Type of wild bird	Wild birds	1	Weighted average of all wild birds	225.20	21.52	15.08	0.00
**248**	vorb	Vorombe	Hawk	Wild birds	1	Weighted average of all wild birds	225.20	21.52	15.08	0.00
**249**	vorkah	Voronkahaka	Type of wild bird	Wild birds	1	Weighted average of all wild birds	225.20	21.52	15.08	0.00
**250**	vorts	Vorontsaina	Type of wild bird	Wild birds	1	Weighted average of all wild birds	225.20	21.52	15.08	0.00

ID	Saturated fatty acids (g)	Monosaturated fatty acids (g)	Polyunsaturated fatty acids (g)	Cholesterol (mg)	Fiber (g)	SUCs (g)	Vitamin A in retinol equivalents (µg)	Vitamin D (µg)	Vitamin E (tocopherol equivalents) (mg)
**1**	0.10	0.00	0.10	0.00	2.60	12.20	3.00	0.00	0.10
**2**	0.07	0.02	0.03	0.00	2.40	14.00	83.00	0.00	0.21
**3**	0.10	0.00	0.10	0.00	2.30	3.30	91.00	0.00	0.00
**4**	0.10	0.00	0.10	0.00	2.30	3.30	91.00	0.00	0.00
**5**	0.10	0.00	0.10	0.00	2.30	3.30	91.00	0.00	0.00
**6**	0.07	0.02	0.03	0.00	2.40	14.00	83.00	0.00	0.21
**7**	0.07	0.02	0.03	0.00	2.40	14.00	83.00	0.00	0.21
**8**	0.10	0.00	0.10	0.00	2.30	3.30	91.00	0.00	0.00
**9**	0.10	0.00	0.10	0.00	2.30	3.30	91.00	0.00	0.00
**10**	0.07	0.02	0.03	0.00	2.40	14.00	83.00	0.00	0.21
**11**	0.07	0.02	0.03	0.00	2.40	14.00	83.00	0.00	0.21
**12**	0.07	0.02	0.03	0.00	2.40	14.00	83.00	0.00	0.21
**13**	0.07	0.02	0.03	0.00	2.40	14.00	83.00	0.00	0.21
**14**	0.10	0.00	0.10	0.00	1.75	3.30	45.50	0.00	0.12
**15**	0.10	0.00	0.30	0.00	6.40	0.30	0.00	0.00	0.00
**16**	0.10	0.00	0.20	0.00	6.50	1.40	3.00	0.00	0.14
**17**	3.06	10.92	6.96	0.00	8.80	2.46	0.00	0.00	2.58
**18**	0.30	0.60	1.20	0.00	7.50	1.40	3.00	0.00	0.00
**19**	0.10	0.00	0.40	0.00	8.40	1.30	0.00	0.00	0.00
**20**	0.10	0.03	0.17	0.00	7.03	2.10	1.33	0.00	0.16
**21**	0.10	0.00	0.20	0.00	6.50	1.40	79.00	0.00	0.14
**22**	0.09	0.03	0.17	0.00	7.00	2.90	0.00	0.00	0.18
**23**	0.10	0.00	0.20	0.00	6.50	1.40	79.00	0.00	0.14

	Saturated fatty acids (g)	Monosaturated fatty acids (g)	Polyunsaturated fatty acids (g)	Cholesterol (mg)	Fiber (g)	SUCs (g)	Vitamin A in retinol equivalents (µg)	Vitamin D (µg)	Vitamin E (tocopherol equivalents) (mg)
**24**	0.12	0.05	0.13	0.00	7.60	2.00	1.00	0.00	0.15
**25**	7.00	18.80	21.80	0.00	16.90	0.70	1.00	0.00	2.00
**26**	0.10	0.00	0.30	0.00	6.40	0.30	0.00	0.00	0.00
**27**	0.10	0.00	0.20	0.00	6.50	1.40	79.00	0.00	0.14
**28**	1.00	0.50	0.00	5.80	0.00	0.00	22.60	0.00	0.00
**29**	1.90	0.80	0.20	10.00	0.00	5.30	28.00	0.00	0.10
**30**	6.30	0.50	0.20	0.00	1.00	13.40	0.00	0.00	0.10
**31**	1.10	1.70	4.40	0.00	1.90	0.30	0.00	0.00	0.00
**32**	0.29	0.28	0.83	0.00	4.60	1.56	0.00	0.00	0.45
**33**	0.20	0.20	0.20	0.00	3.60	2.20	14.00	0.00	0.00
**34**	1.10	1.70	4.40	0.00	1.90	0.30	0.00	0.00	0.00
**35**	1.50	3.11	1.11	51.90	3.80	1.63	0.00	0.00	0.36
**36**	1.50	3.11	1.11	51.90	3.80	1.63	0.00	0.00	0.36
**37**	1.25	0.15	0.15	0.00	1.75	1.70	45.50	0.00	0.12
**38**	1.50	3.11	1.11	51.90	3.80	1.63	0.00	0.00	0.36
**39**	1.30	1.71	0.64	77.00	0.00	0.00	0.00	0.00	0.38
**40**	1.30	1.71	0.64	77.00	0.00	0.00	0.00	0.00	0.38
**41**	1.30	1.71	0.64	77.00	0.00	0.00	0.00	0.00	0.38
**42**	1.30	1.71	0.64	77.00	0.00	0.00	0.00	0.00	0.38
**43**	1.30	1.71	0.64	77.00	0.00	0.00	0.00	0.00	0.38
**44**	1.30	1.71	0.64	77.00	0.00	0.00	0.00	0.00	0.38
**45**	1.30	1.71	0.64	77.00	0.00	0.00	0.00	0.00	0.38
**46**	1.30	1.71	0.64	77.00	0.00	0.00	0.00	0.00	0.38
**47**	1.30	1.71	0.64	77.00	0.00	0.00	0.00	0.00	0.38
**48**	1.30	1.71	0.64	77.00	0.00	0.00	0.00	0.00	0.38
**49**	1.30	1.71	0.64	77.00	0.00	0.00	0.00	0.00	0.38
**50**	1.30	1.71	0.64	77.00	0.00	0.00	0.00	0.00	0.38
**51**	1.30	1.71	0.64	77.00	0.00	0.00	0.00	0.00	0.38
**52**	1.30	1.71	0.64	77.00	0.00	0.00	0.00	0.00	0.38

	Saturated fatty acids (g)	Monosaturated fatty acids (g)	Polyunsaturated fatty acids (g)	Cholesterol (mg)	Fiber (g)	SUCs (g)	Vitamin A in retinol equivalents (µg)	Vitamin D (µg)	Vitamin E (tocopherol equivalents) (mg)
**53**	1.30	1.71	0.64	77.00	0.00	0.00	0.00	0.00	0.38
**54**	0.08	0.05	0.10	50.00	0.00	0.00	15.00	8.00	1.00
**55**	1.30	1.71	0.64	77.00	0.00	0.00	0.00	0.00	0.38
**56**	1.30	1.71	0.64	77.00	0.00	0.00	0.00	0.00	0.38
**57**	1.30	1.71	0.64	77.00	0.00	0.00	0.00	0.00	0.38
**58**	1.30	1.71	0.64	77.00	0.00	0.00	0.00	0.00	0.38
**59**	1.30	1.71	0.64	77.00	0.00	0.00	0.00	0.00	0.38
**60**	1.30	1.71	0.64	77.00	0.00	0.00	0.00	0.00	0.38
**61**	11.40	10.10	0.90	82.00	0.00	0.00	0.00	0.10	0.33
**62**	19.30	22.50	5.40	101.00	0.00	0.30	0.00	1.20	0.10
**63**	10.40	9.00	0.80	61.00	0.00	0.00	0.00	0.50	0.29
**64**	30.26	25.34	1.70	35.00	0.00	0.00	0.00	0.30	0.00
**65**	9.67	12.90	3.65	100.00	0.00	0.00	63.00	0.10	0.70
**66**	2.16	2.65	2.12	90.00	0.00	0.00	28.00	0.50	0.07
**67**	9.67	12.90	3.65	100.00	0.00	0.00	63.00	3.00	0.70
**68**	9.67	12.90	3.65	100.00	0.00	0.00	63.00	3.00	0.70
**69**	2.30	3.61	1.51	86.00	0.00	0.00	35.00	0.10	0.22
**70**	2.00	3.60	2.00	57.00	0.00	0.00	0.00	2.00	0.00
**71**	0.46	0.35	0.93	189.00	0.00	0.00	5.00	0.00	7.30
**72**	0.46	0.35	0.93	189.00	0.00	0.00	5.00	0.00	7.30
**73**	0.70	0.40	1.10	193.00	0.00	0.00	49.00	4.00	4.00
**74**	0.46	0.35	0.93	189.00	0.00	0.00	5.00	0.00	7.30
**75**	0.70	0.40	1.10	193.00	0.00	0.00	49.00	4.00	4.00
**76**	13.20	4.50	1.50	395.70	0.00	0.00	114.00	0.60	1.30
**77**	3.68	6.53	1.22	884.00	0.00	0.93	194.00	1.70	1.34
**78**	0.60	0.90	0.50	58.00	0.00	0.00	12.00	10.00	2.00
**79**	3.02	9.22	1.21	161.00	0.00	0.00	322.00	10.00	2.00
**80**	0.60	0.90	0.50	58.00	0.00	0.00	12.00	10.00	2.00
**81**	0.65	1.07	0.51	60.06	0.00	0.00	18.20	10.00	2.00

	Saturated fatty acids (g)	Monosaturated fatty acids (g)	Polyunsaturated fatty acids (g)	Cholesterol (mg)	Fiber (g)	SUCs (g)	Vitamin A in retinol equivalents (µg)	Vitamin D (µg)	Vitamin E (tocopherol equivalents) (mg)
**82**	0.60	0.90	0.50	58.00	0.00	0.00	12.00	10.00	2.00
**83**	0.00	0.00	0.00	0.00	0.00	0.00	0.00	0.00	0.00
**84**	0.10	0.00	0.10	0.00	1.60	6.50	15.00	0.00	0.00
**85**	0.00	0.00	0.00	0.00	1.10	7.30	2.00	0.00	0.10
**86**	0.10	0.10	0.10	0.00	3.30	13.50	0.00	0.00	0.10
**87**	0.30	0.10	0.40	0.00	5.40	8.90	60.00	0.00	0.70
**88**	0.10	0.12	0.13	0.00	1.00	15.23	0.00	0.00	0.07
**89**	0.01	0.05	0.06	0.00	3.60	7.05	1.00	0.00	0.12
**90**	0.00	0.00	0.00	0.00	1.40	9.30	3.00	0.00	0.00
**91**	0.10	0.10	0.10	0.00	1.80	9.90	168.00	0.00	0.00
**92**	0.00	0.00	0.10	0.00	2.40	10.40	3.00	0.00	0.20
**93**	0.10	0.00	0.20	0.00	2.80	0.50	13.00	0.00	0.00
**94**	0.00	0.00	0.00	0.00	1.80	1.80	135.00	0.00	1.00
**95**	0.00	0.00	0.00	0.00	2.40	4.20	8.00	0.00	0.00
**96**	0.87	4.03	0.76	0.00	17.70	4.42	24.10	0.00	0.96
**97**	0.20	0.10	0.20	0.00	2.50	14.70	2.00	0.00	0.96
**98**	2.10	9.80	1.80	0.00	6.70	0.70	7.00	0.00	2.10
**99**	0.10	0.10	0.10	0.00	1.80	9.90	168.00	0.00	0.00
**100**	0.87	4.03	0.76	0.00	3.72	4.42	24.10	0.00	0.96
**101**	0.87	4.03	0.76	0.00	3.72	4.42	24.10	0.00	0.96
**102**	0.03	0.03	0.08	0.00	1.20	2.63	42.00	0.00	0.54
**103**	0.87	4.03	0.76	0.00	3.72	4.42	24.10	0.00	0.96
**104**	0.87	4.03	0.76	0.00	3.72	4.42	24.10	0.00	0.96
**105**	0.87	4.03	0.76	0.00	3.72	4.42	24.10	0.00	0.96
**106**	0.87	4.03	0.76	0.00	3.72	4.42	24.10	0.00	0.96
**107**	0.00	0.00	0.00	0.00	2.40	4.20	8.00	0.00	0.00
**108**	0.87	4.03	0.76	0.00	3.72	4.42	24.10	0.00	0.96
**109**	0.87	4.03	0.76	0.00	3.72	4.42	24.10	0.00	0.96
**110**	0.87	4.03	0.76	0.00	3.72	4.42	24.10	0.00	0.96

	Saturated fatty acids (g)	Monosaturated fatty acids (g)	Polyunsaturated fatty acids (g)	Cholesterol (mg)	Fiber (g)	SUCs (g)	Vitamin A in retinol equivalents (µg)	Vitamin D (µg)	Vitamin E (tocopherol equivalents) (mg)
**111**	0.87	4.03	0.76	0.00	3.72	4.42	24.10	0.00	0.96
**112**	0.00	0.00	0.00	0.00	1.80	1.80	135.00	0.00	1.00
**113**	0.15	0.47	0.02	0.00	2.00	1.00	351.00	0.00	0.10
**114**	0.02	0.24	0.23	0.00	3.80	0.70	346.00	0.00	0.70
**115**	0.00	0.10	0.00	0.00	2.00	0.00	105.00	0.00	2.00
**116**	0.03	0.01	0.08	0.00	1.90	1.04	87.00	0.00	1.45
**117**	0.09	0.08	0.15	0.00	2.03	0.48	146.00	0.00	0.92
**118**	0.21	0.05	0.02	0.00	2.40	0.48	97.00	0.00	0.96
**119**	0.00	0.00	0.10	0.00	1.50	5.00	248.62	0.00	0.92
**120**	0.20	0.10	0.30	0.00	3.70	3.00	424.00	0.00	2.00
**121**	0.08	0.13	0.11	0.00	2.03	0.48	248.62	0.00	0.92
**122**	0.10	0.00	0.10	0.00	4.20	0.00	72.00	0.00	0.00
**123**	0.08	0.13	0.11	0.00	2.03	0.48	248.62	0.00	0.92
**124**	0.00	0.00	0.10	0.00	0.60	0.00	46.00	0.00	0.00
**125**	0.03	0.02	0.10	0.00	1.00	1.18	223.00	0.00	0.09
**126**	0.08	0.13	0.11	0.00	2.03	0.48	248.62	0.00	0.92
**127**	0.08	0.13	0.11	0.00	2.03	0.48	248.62	0.00	0.92
**128**	0.08	0.13	0.11	0.00	2.03	0.48	248.62	0.00	0.92
**129**	0.08	0.13	0.11	0.00	2.03	0.48	248.62	0.00	0.92
**130**	0.08	0.13	0.11	0.00	2.03	0.48	248.62	0.00	0.92
**131**	0.08	0.13	0.11	0.00	2.03	0.48	248.62	0.00	0.92
**132**	0.08	0.13	0.11	0.00	2.03	0.48	248.62	0.00	0.92
**133**	0.08	0.13	0.11	0.00	2.03	0.48	248.62	0.00	0.92
**134**	0.08	0.13	0.11	0.00	2.03	0.48	248.62	0.00	0.92
**135**	0.08	0.13	0.11	0.00	2.03	0.48	248.62	0.00	0.92
**136**	0.08	0.13	0.11	0.00	2.03	0.48	181.00	0.00	0.92
**137**	0.10	0.00	0.20	0.00	2.80	0.50	13.00	0.00	0.00
**138**	0.08	0.13	0.11	0.00	2.03	0.48	248.62	0.00	0.92

	Saturated fatty acids (g)	Monosaturated fatty acids (g)	Polyunsaturated fatty acids (g)	Cholesterol (mg)	Fiber (g)	SUCs (g)	Vitamin A in retinol equivalents (µg)	Vitamin D (µg)	Vitamin E (tocopherol equivalents) (mg)
**139**	0.08	0.13	0.11	0.00	2.03	0.48	248.62	0.00	0.92
**140**	0.08	0.13	0.11	0.00	2.03	0.48	248.62	0.00	0.92
**141**	0.08	0.13	0.11	0.00	2.03	0.48	248.62	0.00	0.92
**142**	0.07	0.02	0.12	0.00	3.20	0.81	579.00	0.00	2.86
**143**	0.08	0.13	0.11	0.00	2.03	0.48	248.62	0.00	0.92
**144**	0.08	0.13	0.11	0.00	2.03	0.48	248.62	0.00	0.92
**145**	0.08	0.13	0.11	0.00	2.03	0.48	248.62	0.00	0.92
**146**	0.24	0.05	0.52	75	6.40	0	12.54	1	0
**147**	0.00	0.00	0.00	0.00	0.00	1.50	0.00	0.00	0.00
**148**	0.24	0.05	0.52	75	6.40	0	12.54	1	0
**149**	0.24	0.05	0.52	75	6.40	0	12.54	1	0
**150**	0.24	0.05	0.52	75	6.40	0	12.54	1	0
**151**	2.149	2.069	1.5541	75	6.40	0	8.58	1	0
**152**	3.00	0.10	0.00	0.00	0.90	7.40	0.00	0.00	0.00
**153**	3.06	10.92	6.96	0.00	8.80	2.46	0.00	0.00	2.58
**154**	0.05	0.01	0.09	0.00	1.50	17.16	3.00	0.00	0.50
**155**	0.05	0.01	0.09	0.00	1.50	17.16	3.00	0.00	0.50
**156**	0.05	0.01	0.09	0.00	1.50	17.16	3.00	0.00	0.50
**157**	0.05	0.01	0.09	0.00	1.50	17.16	3.00	0.00	0.50
**158**	0.05	0.01	0.09	0.00	1.50	17.16	3.00	0.00	0.50
**159**	0.05	0.01	0.09	0.00	1.50	17.16	3.00	0.00	0.50
**160**	0.05	0.01	0.09	0.00	1.50	17.16	3.00	0.00	0.50
**161**	0.05	0.01	0.09	0.00	1.50	17.16	3.00	0.00	0.50
**162**	0.05	0.01	0.09	0.00	1.50	17.16	3.00	0.00	0.50
**163**	0.05	0.01	0.09	0.00	1.50	17.16	3.00	0.00	0.50
**164**	0.05	0.01	0.09	0.00	1.50	17.16	3.00	0.00	0.50
**165**	0.05	0.01	0.09	0.00	1.50	17.16	3.00	0.00	0.50
**166**	0.05	0.01	0.09	0.00	1.50	17.16	3.00	0.00	0.50

	Saturated fatty acids (g)	Monosaturated fatty acids (g)	Polyunsaturated fatty acids (g)	Cholesterol (mg)	Fiber (g)	SUCs (g)	Vitamin A in retinol equivalents (µg)	Vitamin D (µg)	Vitamin E (tocopherol equivalents) (mg)
**167**	0.05	0.01	0.09	0.00	1.50	17.16	3.00	0.00	0.50
**168**	0.05	0.01	0.09	0.00	1.50	17.16	3.00	0.00	0.50
**169**	0.05	0.01	0.09	0.00	1.50	17.16	3.00	0.00	0.50
**170**	0.05	0.01	0.09	0.00	1.50	17.16	3.00	0.00	0.50
**171**	0.05	0.01	0.09	0.00	1.50	17.16	3.00	0.00	0.50
**172**	0.05	0.01	0.09	0.00	1.50	17.16	3.00	0.00	0.50
**173**	0.05	0.01	0.09	0.00	1.50	17.16	3.00	0.00	0.50
**174**	0.05	0.01	0.09	0.00	1.50	17.16	3.00	0.00	0.50
**175**	0.05	0.01	0.09	0.00	1.50	17.16	3.00	0.00	0.50
**176**	0.05	0.01	0.09	0.00	1.50	17.16	3.00	0.00	0.50
**177**	0.05	0.01	0.09	0.00	1.50	17.16	3.00	0.00	0.50
**178**	0.05	0.01	0.09	0.00	1.50	17.16	3.00	0.00	0.50
**179**	0.05	0.01	0.09	0.00	1.50	17.16	3.00	0.00	0.50
**180**	0.05	0.01	0.09	0.00	1.50	17.16	3.00	0.00	0.50
**181**	0.05	0.01	0.09	0.00	1.50	17.16	3.00	0.00	0.50
**182**	0.41	0.51	0.52	41.00	5.30	0.00	17.00	0.00	0.20
**183**	0.41	0.51	0.52	41.00	5.30	0.00	17.00	0.00	0.20
**184**	2.40	0.30	0.20	0.00	1.20	0.10	0.00	0.00	0.23
**185**	2.40	0.30	0.20	0.00	1.20	0.10	0.00	0.00	0.23
**186**	2.40	0.30	0.20	0.00	1.20	0.10	0.00	0.00	0.23
**187**	0.50	0.80	0.60	45.00	0.00	0.00	0.00	0.00	0.00
**188**	0.06	0.05	0.08	189.00	0.00	0.00	54.00	0.00	1.10
**189**	0.45	0.32	0.48	96.00	0.00	0.00	90.00	0.00	1.20
**190**	0.06	0.05	0.08	189.00	0.00	0.00	54.00	0.10	1.10
**191**	0.30	0.80	0.70	33.00	0.00	0.00	43.00	12.00	1.00
**192**	0.00	0.00	0.20	0.00	2.10	2.00	0.00	0.00	0.00
**193**	0.90	0.00	0.20	0.00	2.10	1.00	0.00	0.00	0.00
**194**	0.20	0.20	0.20	0.00	3.60	2.20	14.00	0.00	0.00
**195**	0.00	0.00	0.10	0.00	4.30	0.40	2.00	0.00	0.49

	Saturated fatty acids (g)	Monosaturated fatty acids (g)	Polyunsaturated fatty acids (g)	Cholesterol (mg)	Fiber (g)	SUCs (g)	Vitamin A in retinol equivalents (µg)	Vitamin D (µg)	Vitamin E (tocopherol equivalents) (mg)
**196**	0.00	0.00	0.10	0.00	4.10	0.40	3.00	0.00	2.40
**197**	0.00	0.00	0.00	0.00	5.10	0.50	4.00	0.00	2.40
**198**	0.00	0.00	0.10	0.00	4.90	11.00	4.00	0.00	1.00
**199**	0.00	0.00	0.00	0.00	3.00	2.80	787.00	0.00	5.00
**200**	0.05	0.05	0.08	0.00	3.50	3.08	166.00	0.00	1.76
**201**	0.00	0.00	0.10	0.00	4.30	0.40	2.00	0.00	0.49
**202**	0.24	0.13	0.63	0.00	3.00	0.49	0.00	0.00	0.06
**203**	3.43	5.37	4.49	13.00	0.00	14.47	0.00	0.00	0.00
**204**	0.00	0.00	0.00	0.00	0.00	0.00	0.00	0.00	0.00
**205**	81.50	11.40	1.60	0.00	0.00	0.00	0.00	0.00	4.00
**206**	13.80	0.00	0.30	0.30	0.00	3.30	261.00	0.00	0.00
**207**	0.00	0.00	0.00	0.00	0.00	0.00	0.00	0.00	0.00
**208**	0.00	0.00	0.00	0.00	0.00	96.80	0.00	0.00	0.00
**209**	0.00	0.00	0.00	0.00	0.00	1.50	0.00	0.00	0.00
**210**	0.00	0.00	0.00	0.00	0.00	5.90	0.00	0.00	0.00
**211**	0.00	0.00	0.00	0.00	0.00	12.60	0.00	0.00	0.00
**212**	5.30	2.60	0.20	33.00	0.00	41.60	65.00	0.10	0.16
**213**	0.00	0.00	0.10	0.00	1.30	1.20	0.00	0.00	0.00
**214**	0.10	0.00	0.20	0.00	2.80	0.50	13.00	0.00	0.00
**215**	0.00	0.00	0.00	0.00	0.50	1.70	5.00	0.00	0.00
**216**	0.00	0.00	0.10	0.00	2.80	4.70	841.00	0.00	0.70
**217**	0.01	0.03	0.08	0.00	2.80	1.95	24.00	0.00	0.14
**218**	0.10	0.00	0.20	0.00	2.20	0.20	0.00	2.00	0.00
**219**	0.04	0.01	0.08	0.00	1.00	1.76	70.00	0.00	0.02
**220**	0.04	0.01	0.08	0.00	1.00	1.76	70.00	0.00	0.02
**221**	0.00	0.00	0.00	0.00	2.80	1.89	0.00	0.00	0.14
**222**	0.20	0.37	0.60	0.00	2.40	4.54	13.00	0.00	0.09
**223**	0.00	0.00	0.00	0.00	3.00	2.80	29.00	0.00	0.00

	Saturated fatty acids (g)	Monosaturated fatty acids (g)	Polyunsaturated fatty acids (g)	Cholesterol (mg)	Fiber (g)	SUCs (g)	Vitamin A in retinol equivalents (µg)	Vitamin D (µg)	Vitamin E (tocopherol equivalents) (mg)
**224**	0.00	0.00	0.10	0.00	0.40	7.60	20.00	0.00	0.00
**225**	0.00	0.00	0.00	0.00	0.50	1.70	5.00	0.00	0.00
**226**	0.00	0.00	0.00	0.00	2.80	1.89	0.00	0.00	0.14
**227**	3.74	5.46	2.73	116.00	0.00	0.00	28.00	0.20	0.06
**228**	1.77	2.43	1.41	74.00	0.00	0.00	28.00	0.00	0.43
**229**	4.77	6.24	2.50	89.60	0.00	0.00	50.00	6.25	0.43
**230**	4.77	6.24	2.50	89.60	0.00	0.00	50.00	6.25	0.43
**231**	4.77	6.24	2.50	89.60	0.00	0.00	50.00	6.25	0.43
**232**	4.77	6.24	2.50	89.60	0.00	0.00	50.00	6.25	0.43
**233**	4.77	6.24	2.50	89.60	0.00	0.00	50.00	6.25	0.43
**234**	4.77	6.24	2.50	89.60	0.00	0.00	50.00	6.25	0.43
**235**	4.77	6.24	2.50	89.60	0.00	0.00	50.00	6.25	0.43
**236**	4.77	6.24	2.50	89.60	0.00	0.00	50.00	6.25	0.43
**237**	4.77	6.24	2.50	89.60	0.00	0.00	50.00	6.25	0.43
**238**	4.77	6.24	2.50	89.60	0.00	0.00	50.00	6.25	0.43
**239**	4.77	6.24	2.50	89.60	0.00	0.00	50.00	6.25	0.43
**240**	4.77	6.24	2.50	89.60	0.00	0.00	50.00	6.25	0.43
**241**	4.77	6.24	2.50	89.60	0.00	0.00	50.00	6.25	0.43
**242**	4.77	6.24	2.50	89.60	0.00	0.00	50.00	6.25	0.43
**243**	4.77	6.24	2.50	89.60	0.00	0.00	50.00	6.25	0.43
**244**	4.77	6.24	2.50	89.60	0.00	0.00	50.00	6.25	0.43
**245**	4.77	6.24	2.50	89.60	0.00	0.00	50.00	6.25	0.43
**246**	4.77	6.24	2.50	89.60	0.00	0.00	50.00	6.25	0.43
**247**	4.77	6.24	2.50	89.60	0.00	0.00	50.00	6.25	0.43
**248**	4.77	6.24	2.50	89.60	0.00	0.00	50.00	6.25	0.43
**249**	4.77	6.24	2.50	89.60	0.00	0.00	50.00	6.25	0.43
**250**	4.77	6.24	2.50	89.60	0.00	0.00	50.00	6.25	0.43

ID	Vitamin C (mg)	Thiamin (B1) (mg)	Riboflavin (B2) (mg)	Niacin (mg)	Vitamin B6 (mg)	Folate (µg)	Vitamin B12 (µg)	CA (mg)
**1**	8.70	0.00	0.10	0.70	0.40	20.00	0.00	5.00
**2**	8.00	0.04	0.04	0.50	0.22	12.00	0.00	7.00
**3**	11.00	0.10	0.10	0.80	0.20	26.00	0.00	2.00
**4**	11.00	0.10	0.10	0.80	0.20	26.00	0.00	2.00
**5**	11.00	0.10	0.10	0.80	0.20	26.00	0.00	2.00
**6**	8.00	0.04	0.04	0.50	0.22	12.00	0.00	7.00
**7**	8.00	0.04	0.04	0.50	0.22	12.00	0.00	7.00
**8**	11.00	0.10	0.10	0.80	0.20	26.00	0.00	2.00
**9**	11.00	0.10	0.10	0.80	0.20	26.00	0.00	2.00
**10**	8.00	0.04	0.04	0.50	0.22	12.00	0.00	7.00
**11**	8.00	0.04	0.04	0.50	0.22	12.00	0.00	7.00
**12**	8.00	0.04	0.04	0.50	0.22	12.00	0.00	7.00
**13**	8.00	0.04	0.04	0.50	0.22	12.00	0.00	7.00
**14**	5.50	0.09	0.06	1.10	0.15	18.50	0.00	5.00
**15**	1.20	0.20	0.10	0.60	0.10	130.00	0.00	28.00
**16**	0.00	0.15	0.04	0.80	0.15	73.00	0.00	29.00
**17**	10.00	0.26	0.06	5.26	0.15	75.00	0.00	55.00
**18**	1.00	0.10	0.10	0.50	0.10	172.00	0.00	49.00
**19**	1.00	0.20	0.10	0.80	0.20	170.00	0.00	37.00
**20**	0.33	0.16	0.05	0.60	0.13	105.00	0.00	24.33
**21**	2.20	0.15	0.04	0.80	0.15	73.00	0.00	29.00
**22**	0.00	0.16	0.06	0.42	0.16	83.00	0.00	17.00
**23**	2.20	0.15	0.04	0.80	0.15	73.00	0.00	29.00

	Vitamin C (mg)	Thiamin (B1) (mg)	Riboflavin (B2) (mg)	Niacin (mg)	Vitamin B6 (mg)	Folate (µg)	Vitamin B12 (µg)	CA (mg)
**24**	1.00	0.16	0.06	0.58	0.07	159.00	0.00	27.00
**25**	0.00	0.80	0.30	4.50	0.80	97.00	0.00	975.00
**26**	1.20	0.20	0.10	0.60	0.10	130.00	0.00	28.00
**27**	2.20	0.15	0.04	0.80	0.15	73.00	0.00	29.00
**28**	0.40	0.00	0.10	0.00	0.00	2.40	0.20	0.10
**29**	0.00	0.00	0.20	0.10	0.00	5.00	0.40	115.00
**30**	0.10	0.10	0.00	0.40	0.10	3.30	0.00	3.40
**31**	0.00	0.10	0.00	0.80	0.00	13.00	0.00	11.00
**32**	0.00	0.43	0.29	3.36	0.09	81.00	0.00	178.00
**33**	72.00	0.30	0.10	1.40	0.70	36.00	0.00	46.00
**34**	0.00	0.10	0.00	0.80	0.00	13.00	0.00	11.00
**35**	0.00	0.43	0.27	4.07	0.08	92.00	0.00	176.00
**36**	0.00	0.43	0.27	4.07	0.08	92.00	0.00	176.00
**37**	5.50	0.09	0.06	1.10	0.15	18.50	0.00	5.00
**38**	0.00	0.43	0.27	4.07	0.08	92.00	0.00	176.00
**39**	0.00	0.31	0.14	4.21	0.42	6.00	0.70	1.91
**40**	0.00	0.31	0.14	4.21	0.42	6.00	0.70	19.64
**41**	0.00	0.31	0.14	4.21	0.42	6.00	0.70	26.27
**42**	0.00	0.31	0.14	4.21	0.42	6.00	0.70	13.90
**43**	0.00	0.31	0.14	4.21	0.42	6.00	0.70	1.91
**44**	0.00	0.31	0.14	4.21	0.42	6.00	0.70	41.35
**45**	0.00	0.31	0.14	4.21	0.42	6.00	0.70	19.64
**46**	0.00	0.31	0.14	4.21	0.42	6.00	0.70	13.90
**47**	0.00	0.31	0.14	4.21	0.42	6.00	0.70	1.91
**48**	0.00	0.31	0.14	4.21	0.42	6.00	0.70	13.90
**49**	0.00	0.31	0.14	4.21	0.42	6.00	0.70	15.43
**50**	0.00	0.31	0.14	4.21	0.42	6.00	0.70	15.43
**51**	0.00	0.31	0.14	4.21	0.42	6.00	0.70	15.43
**52**	0.00	0.31	0.14	4.21	0.42	6.00	0.70	15.43
**53**	0.00	0.31	0.14	4.21	0.42	6.00	0.70	16.00

	Vitamin C (mg)	Thiamin (B1) (mg)	Riboflavin (B2) (mg)	Niacin (mg)	Vitamin B6 (mg)	Folate (µg)	Vitamin B12 (µg)	CA (mg)
**54**	0.00	0.14	0.25	1.20	0.12	15.00	0.40	18.00
**55**	0.00	0.31	0.14	4.21	0.42	6.00	0.70	15.43
**56**	0.00	0.31	0.14	4.21	0.42	6.00	0.70	13.90
**57**	0.00	0.31	0.14	4.21	0.42	6.00	0.70	13.90
**58**	0.00	0.31	0.14	4.21	0.42	6.00	0.70	13.90
**59**	0.00	0.31	0.14	4.21	0.42	6.00	0.70	1.91
**60**	0.00	0.31	0.14	4.21	0.42	6.00	0.70	15.43
**61**	0.00	0.04	0.17	3.50	0.21	5.00	1.80	13.00
**62**	0.00	0.37	0.18	2.70	0.26	3.00	0.60	13.00
**63**	0.00	0.04	0.26	5.20	0.15	8.00	1.40	7.00
**64**	0.00	0.06	0.03	1.27	0.06	4.00	0.34	17.00
**65**	0.00	0.17	0.27	4.83	0.18	6.00	0.30	11.00
**66**	0.00	0.05	0.28	9.57	0.62	9.00	1.02	14.00
**67**	0.00	0.17	0.27	4.83	0.18	6.00	0.30	11.00
**68**	0.00	0.17	0.27	4.83	0.18	6.00	0.30	11.00
**69**	0.00	0.04	0.18	4.10	0.20	5.00	0.20	12.00
**70**	0.00	0.10	0.30	8.10	0.40	28.00	12.00	1700.00
**71**	0.00	0.03	0.07	2.80	0.00	70.00	5.00	1200.00
**72**	0.00	0.03	0.07	0.10	0.00	70.00	5.00	770.00
**73**	4.00	0.30	0.30	8.80	1.00	42.00	3.70	49.00
**74**	0.00	0.03	0.07	0.10	0.00	70.00	5.00	770.00
**75**	4.00	0.30	0.30	8.80	1.00	42.00	3.70	49.00
**76**	0.00	0.00	0.30	0.10	0.10	26.40	0.70	47.30
**77**	0.00	0.16	0.40	0.20	0.25	80.00	5.40	64.00
**78**	1.00	0.20	0.10	1.90	0.10	8.00	2.30	9.00
**79**	1.80	0.00	0.99	2.10	0.08	17.00	2.89	23.00
**80**	1.00	0.20	0.10	1.90	0.10	8.00	2.30	9.00
**81**	1.02	0.14	0.20	1.90	0.10	8.18	2.31	188.00
**82**	1.00	0.20	0.10	1.90	0.10	8.00	2.30	9.00
**83**	0.00	0.00	0.00	0.00	0.00	0.00	0.00	0.00

	Vitamin C (mg)	Thiamin (B1) (mg)	Riboflavin (B2) (mg)	Niacin (mg)	Vitamin B6 (mg)	Folate (µg)	Vitamin B12 (µg)	CA (mg)
**84**	6.70	0.00	0.10	0.40	0.10	14.00	0.00	34.00
**85**	33.30	0.00	0.00	0.30	0.00	10.00	0.00	12.00
**86**	20.60	0.10	0.10	0.90	0.10	14.00	0.00	14.00
**87**	184.00	0.10	0.00	1.10	0.10	49.00	0.00	18.00
**88**	25.00	0.02	0.01	0.70	0.05	133.00	0.00	2.00
**89**	5.00	0.03	0.06	0.40	0.02	8.00	0.00	10.00
**90**	36.20	0.10	0.00	0.50	0.10	15.00	0.00	7.00
**91**	15.00	0.00	0.00	0.30	0.10	6.00	0.00	10.00
**92**	4.60	0.00	0.00	0.10	0.00	3.00	0.00	6.00
**93**	20.00	0.10	0.10	0.30	0.10	20.00	0.00	31.00
**94**	62.00	0.00	0.00	0.30	0.00	38.00	0.00	24.00
**95**	53.00	0.10	0.00	0.30	0.10	30.00	0.00	40.00
**96**	23.66	0.08	0.04	0.96	0.17	45.50	0.00	875.00
**97**	18.10	0.05	0.08	0.80	0.17	45.50	0.00	17.00
**98**	10.00	0.10	0.10	1.70	0.30	81.00	0.00	12.00
**99**	15.00	0.00	0.00	0.30	0.10	6.00	0.00	10.00
**100**	23.66	0.08	0.04	0.96	0.17	45.50	0.00	13.41
**101**	23.66	0.08	0.04	0.96	0.17	45.50	0.00	13.41
**102**	13.70	0.04	0.02	0.59	0.08	15.00	0.00	10.00
**103**	23.66	0.08	0.04	0.96	0.17	45.50	0.00	13.41
**104**	23.66	0.08	0.04	0.96	0.17	45.50	0.00	13.41
**105**	23.66	0.08	0.04	0.96	0.17	45.50	0.00	13.41
**106**	23.66	0.08	0.04	0.96	0.17	45.50	0.00	13.41
**107**	53.00	0.10	0.00	0.30	0.10	30.00	0.00	40.00
**108**	23.66	0.08	0.04	0.96	0.17	45.50	0.00	13.41
**109**	23.66	0.08	0.04	0.96	0.17	45.50	0.00	13.41
**110**	23.66	0.08	0.04	0.96	0.17	45.50	0.00	13.41
**111**	23.66	0.08	0.04	0.96	0.17	45.50	0.00	13.41
**112**	62.00	0.00	0.00	0.30	0.00	38.00	0.00	24.00
**113**	31.00	0.22	0.51	2.00	0.93	23.00	0.00	151.00

	Vitamin C (mg)	Thiamin (B1) (mg)	Riboflavin (B2) (mg)	Niacin (mg)	Vitamin B6 (mg)	Folate (µg)	Vitamin B12 (µg)	CA (mg)
**114**	101.00	0.08	0.26	0.80	0.25	280.00	0.00	85.00
**115**	58.00	0.60	3.10	0.40	0.10	73.00	0.00	59.20
**116**	88.00	0.18	0.36	1.11	0.80	128.00	0.00	84.00
**117**	43.30	0.03	0.16	0.66	0.19	85.00	0.00	215.00
**118**	11.00	0.09	0.13	0.92	0.21	36.00	0.00	39.00
**119**	70.00	0.09	0.19	1.18	0.32	101.67	0.00	166.00
**120**	35.50	0.20	0.50	1.50	0.10	126.00	0.00	137.00
**121**	140.00	0.09	0.19	1.18	0.32	101.67	0.00	166.00
**122**	169.00	0.50	7.60	0.70	0.20	85.00	0.00	96.90
**123**	16.00	0.08	0.18	1.10	0.32	101.67	0.00	58.00
**124**	139.00	0.10	0.20	0.90	0.50	104.00	0.00	211.00
**125**	45.00	0.04	0.07	0.50	0.19	66.00	0.00	105.00
**126**	27.97	0.13	0.40	1.18	0.32	101.67	0.00	111.02
**127**	27.97	0.13	0.40	1.18	0.32	101.67	0.00	111.02
**128**	27.97	0.13	0.40	1.18	0.32	101.67	0.00	111.02
**129**	27.97	0.13	0.40	1.18	0.32	101.67	0.00	111.02
**130**	27.97	0.13	0.40	1.18	0.32	101.67	0.00	111.02
**131**	27.97	0.13	0.40	1.18	0.32	101.67	0.00	111.02
**132**	27.97	0.13	0.40	1.18	0.32	101.67	0.00	111.02
**133**	27.97	0.13	0.40	1.18	0.32	101.67	0.00	111.02
**134**	27.97	0.13	0.40	1.18	0.32	101.67	0.00	111.02
**135**	27.97	0.13	0.40	1.18	0.32	101.67	0.00	111.02
**136**	26.60	0.02	0.21	4.98	0.32	101.67	0.00	32.00
**137**	0.00	0.07	0.06	0.30	0.10	20.00	0.00	28.00
**138**	27.97	0.13	0.40	1.18	0.32	101.67	0.00	111.02
**139**	27.97	0.13	0.40	1.18	0.32	101.67	0.00	111.02
**140**	27.97	0.13	0.40	1.18	0.32	101.67	0.00	111.02
**141**	27.97	0.13	0.40	1.18	0.32	101.67	0.00	111.02
**142**	60.00	0.07	0.10	0.60	0.32	194.00	0.00	190.00

	Vitamin C (mg)	Thiamin (B1) (mg)	Riboflavin (B2) (mg)	Niacin (mg)	Vitamin B6 (mg)	Folate (µg)	Vitamin B12 (µg)	CA (mg)
**143**	27.97	0.13	0.40	1.18	0.32	101.67	0.00	111.02
**144**	27.97	0.13	0.40	1.18	0.32	101.67	0.00	111.02
**145**	27.97	0.13	0.40	1.18	0.32	101.67	0.00	111.02
**146**	5.41	0.52	2.62	4.1	0.3	3	1.6	61.28
**147**	0.50	0.00	0.04	0.12	0.02	2.00	0.00	6.00
**148**	5.41	0.52	2.62	4.1	0.3	3	1.6	61.28
**149**	5.41	0.52	2.62	4.1	0.3	3	1.6	61.28
**150**	5.41	0.52	2.62	4.1	0.3	3	1.6	61.28
**151**	7.59	0.52	0.08	4.1	0.3	3	1.6	45.68
**152**	1.00	0.00	0.00	0.20	0.00	8.00	0.00	126.00
**153**	0.00	0.26	0.06	5.26	0.15	75.00	0.00	55.00
**154**	8.00	0.05	0.18	0.90	0.02	24.00	0.00	18.00
**155**	8.00	0.05	0.18	0.90	0.02	24.00	0.00	18.00
**156**	8.00	0.05	0.18	0.90	0.02	24.00	0.00	18.00
**157**	8.00	0.05	0.18	0.90	0.02	24.00	0.00	18.00
**158**	8.00	0.05	0.18	0.90	0.02	24.00	0.00	18.00
**159**	8.00	0.05	0.18	0.90	0.02	24.00	0.00	18.00
**160**	8.00	0.05	0.18	0.90	0.02	24.00	0.00	18.00
**161**	8.00	0.05	0.18	0.90	0.02	24.00	0.00	18.00
**162**	8.00	0.05	0.18	0.90	0.02	24.00	0.00	18.00
**163**	8.00	0.05	0.18	0.90	0.02	24.00	0.00	18.00
**164**	8.00	0.05	0.18	0.90	0.02	24.00	0.00	18.00
**165**	8.00	0.05	0.18	0.90	0.02	24.00	0.00	18.00
**166**	8.00	0.05	0.18	0.90	0.02	24.00	0.00	18.00
**167**	8.00	0.05	0.18	0.90	0.02	24.00	0.00	18.00
**168**	8.00	0.05	0.18	0.90	0.02	24.00	0.00	18.00
**169**	8.00	0.05	0.18	0.90	0.02	24.00	0.00	18.00
**170**	8.00	0.05	0.18	0.90	0.02	24.00	0.00	18.00
**171**	8.00	0.05	0.18	0.90	0.02	24.00	0.00	18.00

	Vitamin C (mg)	Thiamin (B1) (mg)	Riboflavin (B2) (mg)	Niacin (mg)	Vitamin B6 (mg)	Folate (µg)	Vitamin B12 (µg)	CA (mg)
**172**	8.00	0.05	0.18	0.90	0.02	24.00	0.00	18.00
**173**	8.00	0.05	0.18	0.90	0.02	24.00	0.00	18.00
**174**	8.00	0.05	0.18	0.90	0.02	24.00	0.00	18.00
**175**	8.00	0.05	0.18	0.90	0.02	24.00	0.00	18.00
**176**	8.00	0.05	0.18	0.90	0.02	24.00	0.00	18.00
**177**	8.00	0.05	0.18	0.90	0.02	24.00	0.00	18.00
**178**	8.00	0.05	0.18	0.90	0.02	24.00	0.00	18.00
**179**	8.00	0.05	0.18	0.90	0.02	24.00	0.00	18.00
**180**	8.00	0.05	0.18	0.90	0.02	24.00	0.00	18.00
**181**	8.00	0.05	0.18	0.90	0.02	24.00	0.00	18.00
**182**	0.00	0.17	0.17	1.26	0.04	60.00	0.10	10.00
**183**	0.00	0.17	0.17	1.26	0.04	60.00	0.10	10.00
**184**	0.00	0.07	0.02	1.40	0.10	11.00	0.00	8.00
**185**	0.00	0.07	0.02	1.40	0.10	11.00	0.00	8.00
**186**	0.00	0.07	0.02	1.40	0.10	11.00	0.00	8.00
**187**	17.00	0.10	0.10	0.70	0.50	51.00	0.00	7.00
**188**	1.00	0.03	0.09	3.50	0.50	0.10	1.16	17.00
**189**	8.00	0.06	0.08	3.78	0.65	24.00	36.00	106.00
**190**	2.00	0.03	0.03	2.55	0.50	0.10	1.16	70.00
**191**	0.00	0.10	0.10	5.70	0.30	11.00	1.10	48.00
**192**	8.00	1.00	0.00	0.30	0.20	23.00	0.00	33.00
**193**	31.20	0.20	0.10	0.70	1.20	3.00	0.00	181.00
**194**	72.00	0.30	0.10	1.40	0.70	36.00	0.00	46.00
**195**	9.50	0.27	0.06	0.30	0.26	17.00	0.00	26.00
**196**	5.00	0.10	0.00	0.60	0.30	22.00	0.00	43.00
**197**	5.00	0.10	0.50	1.50	0.30	19.00	0.00	18.00
**198**	29.00	0.10	0.00	0.80	0.30	10.00	0.00	17.00
**199**	15.00	0.10	0.10	0.60	0.20	23.00	0.00	28.00
**200**	20.89	0.16	0.04	0.68	0.28	20.33	0.00	27.00

	Vitamin C (mg)	Thiamin (B1) (mg)	Riboflavin (B2) (mg)	Niacin (mg)	Vitamin B6 (mg)	Folate (µg)	Vitamin B12 (µg)	CA (mg)
**201**	78.00	0.27	0.06	0.30	0.26	17.00	0.00	26.00
**202**	0.00	0.33	0.11	0.89	0.08	26.00	0.00	13.00
**203**	0.00	0.20	0.24	3.30	0.20	32.00	1.00	60.00
**204**	0.00	0.00	0.00	0.00	0.00	0.00	0.00	0.00
**205**	0.00	0.00	0.00	0.00	0.00	0.00	0.00	6.00
**206**	57.00	0.20	0.20	1.80	0.20	45.00	0.00	15.00
**207**	0.50	0.00	0.00	0.10	0.00	1.00	0.00	8.00
**208**	0.00	0.00	0.00	0.00	0.00	0.00	0.00	1.00
**209**	1.00	0.00	0.00	0.10	0.00	2.00	0.00	6.00
**210**	0.00	0.00	0.00	0.00	0.00	0.00	0.00	6.00
**211**	0.00	0.00	0.00	0.00	0.00	0.00	0.00	13.00
**212**	1.90	0.05	0.43	0.20	0.05	8.00	1.90	261.00
**213**	7.40	0.00	0.00	0.10	0.10	19.00	0.00	23.00
**214**	0.00	0.07	0.06	0.30	0.10	20.00	0.00	28.00
**215**	2.80	0.00	0.00	0.10	0.00	7.00	0.00	16.00
**216**	5.90	0.10	0.10	1.00	0.10	19.00	0.00	33.00
**217**	84.00	0.04	0.04	0.40	0.04	72.00	0.00	19.00
**218**	4.00	0.10	0.30	4.50	0.10	18.00	0.00	3.00
**219**	5.29	0.03	0.04	0.30	0.09	19.09	0.00	2.00
**220**	5.29	0.03	0.04	0.30	0.09	19.09	0.00	2.00
**221**	8.00	0.03	0.04	0.42	0.12	18.00	0.00	13.00
**222**	5.50	0.09	0.06	1.68	0.14	23.00	0.00	3.00
**223**	6.00	0.00	0.00	0.50	0.10	20.00	0.00	27.00
**224**	5.00	0.00	0.00	0.20	0.00	3.00	0.00	7.00
**225**	2.80	0.00	0.00	0.10	0.00	7.00	0.00	16.00
**226**	8.00	0.03	0.04	0.42	0.12	18.00	0.00	13.00
**227**	2.90	0.28	0.35	7.60	0.57	6.00	0.41	17.00
**228**	1.30	0.06	0.10	7.67	0.38	5.00	0.34	12.20
**229**	4.18	0.23	0.26	6.26	0.56	9.00	0.52	12.20

	Vitamin C (mg)	Thiamin (B1) (mg)	Riboflavin (B2) (mg)	Niacin (mg)	Vitamin B6 (mg)	Folate (µg)	Vitamin B12 (µg)	CA (mg)
**230**	4.18	0.23	0.26	6.26	0.56	9.00	0.52	12.20
**231**	4.18	0.23	0.26	6.26	0.56	9.00	0.52	12.20
**232**	4.18	0.23	0.26	6.26	0.56	9.00	0.52	12.20
**233**	4.18	0.23	0.26	6.26	0.56	9.00	0.52	12.20
**234**	4.18	0.23	0.26	6.26	0.56	9.00	0.52	12.20
**235**	4.18	0.23	0.26	6.26	0.56	9.00	0.52	12.20
**236**	4.18	0.23	0.26	6.26	0.56	9.00	0.52	12.20
**237**	4.18	0.23	0.26	6.26	0.56	9.00	0.52	12.20
**238**	4.18	0.23	0.26	6.26	0.56	9.00	0.52	12.20
**239**	4.18	0.23	0.26	6.26	0.56	9.00	0.52	12.20
**240**	4.18	0.23	0.26	6.26	0.56	9.00	0.52	12.20
**241**	4.18	0.23	0.26	6.26	0.56	9.00	0.52	12.20
**242**	4.18	0.23	0.26	6.26	0.56	9.00	0.52	12.20
**243**	4.18	0.23	0.26	6.26	0.56	9.00	0.52	12.20
**244**	4.18	0.23	0.26	6.26	0.56	9.00	0.52	12.20
**245**	4.18	0.23	0.26	6.26	0.56	9.00	0.52	12.20
**246**	4.18	0.23	0.26	6.26	0.56	9.00	0.52	12.20
**247**	4.18	0.23	0.26	6.26	0.56	9.00	0.52	12.20
**248**	4.18	0.23	0.26	6.26	0.56	9.00	0.52	12.20
**249**	4.18	0.23	0.26	6.26	0.56	9.00	0.52	12.20
**250**	4.18	0.23	0.26	6.26	0.56	9.00	0.52	12.20

ID	P (mg)	MG (mg)	K (mg)	NA (mg)	FE (mg)	ZN (mg)	CU (mg)	MN (mg)	Sources
**1**	22.00	27.00	358.00	1.00	0.30	0.20	0.10	0.20	2,3
**2**	31.00	23.00	237.00	2.00	0.90	0.10	0.08	0.20	1,3,5
**3**	28.00	32.00	465.00	5.00	0.60	0.10	0.10	0.20	2,3
**4**	28.00	32.00	465.00	5.00	0.60	0.10	0.10	0.20	2,3
**5**	28.00	32.00	465.00	5.00	0.60	0.10	0.10	0.20	2,3
**6**	31.00	23.00	237.00	2.00	0.90	0.10	0.08	0.20	1,2,3,5
**7**	31.00	23.00	237.00	2.00	0.90	0.10	0.08	0.20	1,2,3,5
**8**	28.00	32.00	465.00	5.00	0.60	0.10	0.10	0.20	2,3
**9**	28.00	32.00	465.00	5.00	0.60	0.10	0.10	0.20	2,3
**10**	31.00	23.00	237.00	2.00	0.90	0.10	0.08	0.20	1,2,3,5
**11**	31.00	23.00	237.00	2.00	0.90	0.10	0.08	0.20	1,2,3,5
**12**	31.00	23.00	237.00	2.00	0.90	0.10	0.08	0.20	1,2,3,5
**13**	31.00	23.00	237.00	2.00	0.90	0.10	0.08	0.20	1,2,3,5
**14**	64.50	43.50	271.00	3.50	0.65	0.40	0.11	0.20	2,5
**15**	142.00	45.00	403.00	2.00	2.90	1.10	0.20	0.50	2
**16**	141.00	14.00	528.00	6.00	2.70	2.90	0.02	1.30	2,5
**17**	198.00	102.00	180.00	751.00	1.50	1.83	0.81	1.70	1,6,7
**18**	168.00	48.00	291.00	7.00	2.90	1.50	0.40	1.00	2
**19**	186.00	59.00	528.00	3.00	3.80	1.40	0.30	0.60	2,3
**20**	117.00	35.00	434.00	3.33	2.16	1.56	0.32	1.00	1
**21**	141.00	14.00	528.00	6.00	1.10	1.00	0.02	1.30	1,2,6
**22**	111.00	43.00	508.00	2.00	2.39	0.95	0.25	0.39	1,7
**23**	141.00	14.00	528.00	6.00	1.10	1.00	0.02	1.30	2,5,6

	P (mg)	MG (mg)	K (mg)	NA (mg)	FE (mg)	ZN (mg)	CU (mg)	MN (mg)	Sources
**24**	99.00	48.00	266.00	2.00	1.40	0.84	0.70	1.30	1,2
**25**	629.00	351.00	468.00	11.00	14.60	7.80	4.10	2.50	2
**26**	142.00	45.00	403.00	2.00	2.90	1.10	0.20	0.50	2
**27**	141.00	14.00	528.00	6.00	1.10	1.00	0.02	1.30	2,5,6
**28**	0.10	0.00	0.10	0.10	0.00	0.00	0.00	0.00	2
**29**	92.00	11.00	140.00	55.00	0.10	0.40	0.00	0.00	2,3
**30**	40.80	13.70	44.30	1.10	0.30	0.50	0.10	0.50	2
**31**	76.00	15.00	75.00	1.00	0.80	0.50	0.10	0.50	2
**32**	107.00	37.00	186.00	353.00	2.87	1.07	0.24	0.86	1,7
**33**	168.00	24.00	583.00	5.00	1.90	0.70	0.10	0.30	2
**34**	76.00	15.00	75.00	1.00	0.80	0.50	0.10	0.50	2
**35**	104.00	36.00	115.00	524.00	3.55	0.90	0.20	0.50	1,2
**36**	104.00	36.00	115.00	524.00	3.55	0.90	0.20	0.50	1,2
**37**	64.50	43.50	271.00	3.50	0.65	0.40	0.11	0.25	5
**38**	104.00	36.00	115.00	524.00	3.55	0.90	0.20	0.50	1,2
**39**	120.00	1.91	1.91	60.00	1.91	1.91	1.91	1.91	1,4
**40**	120.00	15.82	220.85	60.00	16.48	2.37	0.30	0.24	1,4
**41**	120.00	17.48	252.73	60.00	3.88	3.55	0.13	0.05	1,4
**42**	120.00	16.80	278.39	60.00	9.94	2.44	0.26	0.14	1,4
**43**	120.00	1.91	1.91	60.00	1.91	1.91	1.91	1.91	1,4
**44**	120.00	13.00	256.06	60.00	4.51	4.53	0.65	0.59	1,4
**45**	120.00	15.82	220.85	60.00	16.48	2.37	0.30	0.24	1,4
**46**	120.00	16.80	278.39	60.00	9.94	2.44	0.26	0.14	1,4
**47**	120.00	1.91	1.91	60.00	1.91	1.91	1.91	1.91	1,4
**48**	120.00	16.80	278.39	60.00	9.94	2.44	0.26	0.14	1,4
**49**	120.00	13.00	188.47	60.00	8.05	2.57	0.65	0.59	1,4
**50**	120.00	13.00	188.47	60.00	8.05	2.57	0.65	0.59	1,4
**51**	120.00	13.00	188.47	60.00	8.05	2.57	0.65	0.59	1,4
**52**	120.00	13.00	188.47	60.00	8.05	2.57	0.65	0.59	1,4
**53**	120.00	27.00	396.00	60.00	1.12	3.01	0.14	0.00	1
**54**	147.00	20.00	285.00	58.00	1.50	1.00	0.25	0.00	1,2

	P (mg)	MG (mg)	K (mg)	NA (mg)	FE (mg)	ZN (mg)	CU (mg)	MN (mg)	Sources
**55**	120.00	13.00	188.47	60.00	8.05	2.57	0.65	0.59	1,4
**56**	120.00	16.80	278.39	60.00	9.94	2.44	0.26	0.14	1,4
**57**	120.00	16.80	278.39	60.00	9.94	2.44	0.26	0.14	1,4
**58**	120.00	16.80	278.39	60.00	9.94	2.44	0.26	0.14	1,4
**59**	120.00	1.91	1.91	60.00	1.91	1.91	1.91	1.91	1,4
**60**	120.00	13.00	188.47	60.00	8.05	2.57	0.65	0.59	1,4
**61**	166.00	17.00	223.00	41.00	3.10	6.65	0.10	0.00	2,9
**62**	153.00	15.00	231.00	42.00	2.80	5.50	0.15	0.00	2,9
**63**	184.00	22.00	300.00	38.00	3.50	6.00	0.14	0.00	2,9
**64**	66.00	7.00	129.00	25.00	0.47	0.43	0.10	0.00	1,2
**65**	156.00	16.00	204.00	59.00	2.70	1.86	0.14	0.00	1,3,7
**66**	223.00	30.00	239.00	103.00	1.09	2.80	0.15	0.00	1,7
**67**	156.00	16.00	204.00	59.00	2.70	1.86	0.14	0.00	1,3
**68**	156.00	16.00	204.00	59.00	2.70	1.86	0.14	0.00	1,3
**69**	125.00	125.00	94.00	38.00	1.00	2.08	0.07	0.00	1,9
**70**	1300.00	140.00	953.00	312.00	2.50	5.20	0.50	2.10	2
**71**	710.00	290.00	500.00	457.00	21.30	3.70	2.40	0.00	1,3,8
**72**	517.00	290.00	365.00	547.00	5.30	3.20	2.40	0.00	1,3,8
**73**	483.00	147.00	854.00	273.00	1.80	2.10	0.10	0.10	2
**74**	517.00	290.00	365.00	547.00	5.30	3.20	2.40	0.00	1,3,8
**75**	483.00	147.00	854.00	273.00	1.80	2.10	0.10	0.10	8
**76**	160.50	9.40	117.70	632.10	1.10	1.00	0.00	0.00	2
**77**	220.00	17.00	222.00	146.00	3.85	1.41	0.06	0.04	1,3,7
**78**	243.00	22.00	335.00	40.00	0.30	0.50	0.00	0.00	2
**79**	139.00	26.00	349.00	64.00	1.60	0.70	0.10	0.00	1,8
**80**	243.00	22.00	335.00	40.00	0.30	0.50	0.00	0.00	2
**81**	1.09	6.02	0.95	3.75	0.02	0.01	0.00	0.00	11,16
**82**	243.00	22.00	335.00	40.00	0.30	0.50	0.00	0.00	2
**83**	0.00	0.00	0.00	0.00	0.00	0.00	0.00	0.00	
**84**	36.00	37.00	303.00	3.00	0.60	0.40	0.20	0.20	2

	P (mg)	MG (mg)	K (mg)	NA (mg)	FE (mg)	ZN (mg)	CU (mg)	MN (mg)	Sources
**85**	8.00	9.00	148.00	0.00	0.06	0.07	0.05	0.01	2
**86**	27.00	21.00	278.00	14.00	0.60	0.10	0.10	0.10	2
**87**	40.00	22.00	417.00	2.00	1.40	0.20	0.20	0.20	2,6
**88**	20.00	12.00	224.00	3.00	0.40	0.21	0.10	0.00	1,3
**89**	10.00	8.00	134.00	14.00	0.60	0.02	0.10	0.00	1,8
**90**	7.00	14.00	113.00	1.00	0.40	0.10	0.10	1.70	2
**91**	11.00	9.00	156.00	2.00	0.10	0.00	0.10	0.00	2
**92**	11.00	5.00	107.00	1.00	0.10	0.00	0.00	0.00	2
**93**	15.00	8.00	97.00	8.00	0.20	0.10	0.00	0.10	2
**94**	5.00	10.00	257.00	3.00	0.10	0.10	0.00	0.00	2
**95**	14.00	10.00	181.00	0.00	0.10	0.10	0.10	0.00	2
**96**	76.80	315.00	1075.00	16.00	0.00	9.60	0.00	0.50	17
**97**	28.30	32.00	250.00	4.00	0.70	0.30	0.10	0.50	6,7
**98**	52.00	29.00	599.00	7.00	0.60	0.60	0.20	0.10	2
**99**	11.00	9.00	156.00	2.00	0.10	0.00	0.10	0.00	2
**100**	28.30	18.94	341.25	4.18	0.41	0.31	0.10	0.50	Weighted average of all fruits
**101**	28.30	18.94	341.25	4.18	0.41	0.31	0.10	0.50	Weighted average of all fruits
**102**	24.00	11.00	237.00	5.00	0.27	0.17	0.10	0.10	1
**103**	28.30	18.94	341.25	4.18	0.41	0.31	0.10	0.50	Weighted average of all fruits
**104**	28.30	18.94	341.25	4.18	0.41	0.31	0.10	0.50	Weighted average of all fruits
**105**	28.30	18.94	341.25	4.18	0.41	0.31	0.10	0.50	Weighted average of all fruits
**106**	28.30	18.94	341.25	4.18	0.41	0.31	0.10	0.50	Weighted average of all fruits
**107**	14.00	10.00	181.00	0.00	0.10	0.10	0.10	0.00	2
**108**	28.30	18.94	341.25	4.18	0.41	0.31	0.10	0.50	Weighted average of all fruits
**109**	28.30	18.94	341.25	4.18	0.41	0.31	0.10	0.50	Weighted average of all fruits
**110**	28.30	18.94	341.25	4.18	0.41	0.31	0.10	0.50	Weighted average of all fruits
**111**	28.30	18.94	341.25	4.18	0.41	0.31	0.10	0.50	Weighted average of all fruits
**112**	5.00	10.00	257.00	3.00	0.10	0.10	0.00	0.00	2
**113**	67.00	34.00	344.00	9.00	2.32	0.49	0.16	0.34	1,3
**114**	76.00	23.00	570.00	48.00	3.00	0.70	2.74	0.34	1,7
**115**	41.00	44.80	315.20	10.60	2.90	0.20	0.10	0.30	2

	P (mg)	MG (mg)	K (mg)	NA (mg)	FE (mg)	ZN (mg)	CU (mg)	MN (mg)	Sources
**116**	99.00	85.00	608.00	11.00	2.04	0.30	2.74	0.34	1
**117**	50.00	55.00	611.00	20.00	2.32	0.90	2.74	0.34	1
**118**	104.00	38.00	436.00	11.00	2.22	0.20	0.13	0.34	1,9
**119**	65.93	39.73	340.00	7.00	2.90	0.02	0.00	32.00	1,11,18
**120**	29.00	22.00	203.00	29.00	1.20	0.20	0.30	0.30	2,6
**121**	65.93	39.73	373.86	19.80	0.77	1.87	2.74	0.34	6,11
**122**	50.00	55.00	380.90	6.60	6.00	0.60	0.20	0.90	2,6
**123**	108.00	39.73	373.86	19.80	2.50	0.91	2.74	0.34	14
**124**	72.00	62.00	550.00	11.00	1.90	0.40	0.20	0.10	2,6
**125**	37.00	19.00	252.00	65.00	0.80	0.19	0.04	0.27	1,7
**126**	65.93	39.73	373.86	19.80	1.82	1.87	2.74	0.34	Weighted average of all greens
**127**	65.93	39.73	373.86	19.80	1.82	1.87	2.74	0.34	Weighted average of all greens
**128**	65.93	39.73	373.86	19.80	1.82	1.87	2.74	0.34	Weighted average of all greens
**129**	65.93	39.73	373.86	19.80	1.82	1.87	2.74	0.34	Weighted average of all greens
**130**	65.93	39.73	373.86	19.80	1.82	1.87	2.74	0.34	Weighted average of all greens
**131**	65.93	39.73	373.86	19.80	1.82	1.87	2.74	0.34	Weighted average of all greens
**132**	65.93	39.73	373.86	19.80	1.82	1.87	2.74	0.34	Weighted average of all greens
**133**	65.93	39.73	373.86	19.80	1.82	1.87	2.74	0.34	Weighted average of all greens
**134**	65.93	39.73	373.86	19.80	1.82	1.87	2.74	0.34	Weighted average of all greens
**135**	65.93	39.73	373.86	19.80	1.82	1.87	2.74	0.34	Weighted average of all greens
**136**	101.00	34.00	370.00	1.00	1.31	0.83	0.23	0.34	1
**137**	47.00	8.00	97.00	8.00	0.20	0.10	0.00	0.10	2
**138**	65.93	39.73	373.86	19.80	1.82	1.87	2.74	0.34	Weighted average of all greens
**139**	65.93	39.73	373.86	19.80	1.82	1.87	2.74	0.34	Weighted average of all greens
**140**	65.93	39.73	373.86	19.80	1.82	1.87	2.74	0.34	Weighted average of all greens
**141**	65.93	39.73	373.86	19.80	1.82	1.87	2.74	0.34	Weighted average of all greens
**142**	42.00	31.00	296.00	40.00	1.10	0.19	2.74	0.34	1
**143**	65.93	39.73	373.86	19.80	1.82	1.87	2.74	0.34	Weighted average of all greens
**144**	65.93	39.73	373.86	19.80	1.82	1.87	2.74	0.34	Weighted average of all greens
**145**	65.93	39.73	373.86	19.80	1.82	1.87	2.74	0.34	Weighted average of all greens

	P (mg)	MG (mg)	K (mg)	NA (mg)	FE (mg)	ZN (mg)	CU (mg)	MN (mg)	Sources
**146**	136.4	6.14	25.44	21.37	18.2	10.1	0.01	0.39	2,13,15
**147**	0.40	2.00	52.00	4.00	0.40	0.20	0.00	0.10	2,13
**148**	136.4	6.14	25.44	21.37	18.2	10.1	0.01	0.39	10
**149**	136.4	6.14	25.44	21.37	18.2	10.1	0.01	0.39	10
**150**	136.4	6.14	25.44	21.37	18.2	10.1	0.01	0.39	10
**151**	130.2	6.62	25.44	21.37	2.31	10.1	0.01	0.39	10
**152**	48.00	3.00	66.00	5.00	0.40	0.10	0.10	0.20	2
**153**	198.00	102.00	180.00	751.00	1.01	1.83	0.10	0.20	1
**154**	140.00	10.00	1806.00	14.00	1.69	3.73	0.64	0.00	1
**155**	140.00	10.00	1806.00	14.00	1.69	3.73	0.64	0.00	1
**156**	140.00	10.00	1806.00	14.00	1.69	3.73	0.64	0.00	1
**157**	140.00	10.00	1806.00	14.00	1.69	3.73	0.64	0.00	1
**158**	140.00	10.00	1806.00	14.00	1.69	3.73	0.64	0.00	1
**159**	140.00	10.00	1806.00	14.00	1.69	3.73	0.64	0.00	1
**160**	140.00	10.00	1806.00	14.00	1.69	3.73	0.64	0.00	1
**161**	140.00	10.00	1806.00	14.00	1.69	3.73	0.64	0.00	1
**162**	140.00	10.00	1806.00	14.00	1.69	3.73	0.64	0.00	1
**163**	140.00	10.00	1806.00	14.00	1.69	3.73	0.64	0.00	1
**164**	140.00	10.00	1806.00	14.00	1.69	3.73	0.64	0.00	1
**165**	140.00	10.00	1806.00	14.00	1.69	3.73	0.64	0.00	1
**166**	140.00	10.00	1806.00	14.00	1.69	3.73	0.64	0.00	1
**167**	140.00	10.00	1806.00	14.00	1.69	3.73	0.64	0.00	1
**168**	140.00	10.00	1806.00	14.00	1.69	3.73	0.64	0.00	1
**169**	140.00	10.00	1806.00	14.00	1.69	3.73	0.64	0.00	1
**170**	140.00	10.00	1806.00	14.00	1.69	3.73	0.64	0.00	1
**171**	140.00	10.00	1806.00	14.00	1.69	3.73	0.64	0.00	1
**172**	140.00	10.00	1806.00	14.00	1.69	3.73	0.64	0.00	1
**173**	140.00	10.00	1806.00	14.00	1.69	3.73	0.64	0.00	1
**174**	140.00	10.00	1806.00	14.00	1.69	3.73	0.64	0.00	1
**175**	140.00	10.00	1806.00	14.00	1.69	3.73	0.64	0.00	1
**176**	140.00	10.00	1806.00	14.00	1.69	3.73	0.64	0.00	1

	P (mg)	MG (mg)	K (mg)	NA (mg)	FE (mg)	ZN (mg)	CU (mg)	MN (mg)	Sources
**177**	140.00	10.00	1806.00	14.00	1.69	3.73	0.64	0.00	1
**178**	140.00	10.00	1806.00	14.00	1.69	3.73	0.64	0.00	1
**179**	140.00	10.00	1806.00	14.00	1.69	3.73	0.64	0.00	1
**180**	140.00	10.00	1806.00	14.00	1.69	3.73	0.64	0.00	1
**181**	140.00	10.00	1806.00	14.00	1.69	3.73	0.64	0.00	1
**182**	52.00	14.00	21.00	83.00	1.16	0.44	0.27	2.16	1,7
**183**	52.00	14.00	21.00	83.00	1.16	0.44	0.27	2.16	1,7
**184**	101.00	55.00	77.00	2.00	0.70	0.70	0.11	0.30	2,5
**185**	101.00	55.00	77.00	2.00	0.70	0.70	0.11	0.30	2,5
**186**	101.00	55.00	77.00	2.00	0.70	0.70	0.11	0.30	2,5
**187**	69.00	21.00	122.00	39.00	0.30	0.30	0.00	0.00	2
**188**	141.00	39.00	259.00	111.00	2.41	1.64	0.00	0.00	1,8
**189**	279.00	60.00	630.00	460.00	9.54	3.36	0.00	0.00	1
**190**	237.00	39.00	259.00	111.00	2.41	1.64	0.00	0.00	1
**191**	228.00	86.00	461.00	55.00	0.90	0.40	0.00	0.00	2
**192**	53.00	17.00	249.00	5.00	3.00	0.30	0.10	0.20	2
**193**	143.00	25.00	401.00	17.00	1.70	1.20	0.30	1.70	2
**194**	168.00	24.00	583.00	5.00	1.90	0.70	0.10	0.30	2
**195**	52.00	12.00	687.00	4.00	0.80	0.60	0.11	0.24	2,9
**196**	84.00	33.00	591.00	11.00	0.68	3.20	0.20	0.40	2,6
**197**	76.00	30.00	484.00	15.00	0.70	0.30	0.20	0.40	2
**198**	30.00	25.00	490.00	2.00	0.50	0.10	0.10	0.10	2
**199**	55.00	20.00	348.00	10.00	0.60	0.30	0.20	0.60	2,6
**200**	65.89	21.56	477.33	7.89	0.73	0.41	0.13	0.29	Weighted average: cassava, yam, sweet potato, taro
**201**	52.00	12.00	687.00	4.00	3.10	0.40	0.11	0.24	2,6,9
**202**	108.00	22.00	167.00	2.00	4.50	1.10	0.10	0.70	2,3
**203**	225.00	50.00	403.00	2400.00	2.20	0.21	0.00	0.00	1,2,9
**204**	0.00	0.00	0.00	0.00	0.00	0.00	0.00	0.00	
**205**	7.00	0.00	0.00	0.00	0.00	0.00	0.00	0.00	2

	P (mg)	MG (mg)	K (mg)	NA (mg)	FE (mg)	ZN (mg)	CU (mg)	MN (mg)	Sources
**206**	72.00	33.00	666.00	27.00	1.50	0.30	0.20	0.30	2
**207**	14.00	10.00	89.00	8.00	0.40	0.10	0.00	0.20	2
**208**	2.00	0.00	2.00	1.00	0.10	0.00	0.00	0.00	2,3
**209**	4.00	2.00	52.00	4.00	0.40	0.20	0.00	0.10	2
**210**	2.00	2.00	24.00	3.00	0.10	0.00	0.00	0.00	2
**211**	3.00	4.00	52.00	6.00	0.30	0.00	0.10	0.10	2
**212**	203.00	24.00	303.00	106.00	0.20	0.77	0.02	0.00	2,9
**213**	29.00	10.00	146.00	4.00	0.20	0.20	0.00	0.10	2
**214**	47.00	8.00	97.00	8.00	0.20	0.10	0.00	0.10	2
**215**	21.00	13.00	147.00	2.00	3.00	0.20	0.00	0.10	2
**216**	35.00	12.00	320.00	69.00	0.30	0.20	0.00	0.10	2
**217**	31.00	17.00	296.00	5.00	0.43	0.80	0.20	0.09	1
**218**	86.00	9.00	318.00	5.00	0.50	0.50	0.30	0.00	2
**219**	89.00	15.27	154.00	0.00	0.70	0.27	0.66	0.14	12
**220**	89.00	15.27	154.00	0.00	0.70	0.27	0.66	0.14	12
**221**	29.00	12.00	173.00	1.00	0.22	0.31	0.10	0.10	1
**222**	77.00	26.00	218.00	1.00	0.45	0.62	0.06	0.34	1
**223**	39.00	24.00	192.00	1.00	0.40	0.40	0.10	0.20	2
**224**	11.00	10.00	112.00	1.00	7.30	10.20	0.00	0.00	2
**225**	21.00	13.00	147.00	2.00	3.00	0.20	0.00	0.10	2
**226**	29.00	12.00	173.00	1.00	0.22	0.31	0.00	0.00	1
**227**	332.00	26.00	256.00	57.00	5.91	3.83	0.14	0.00	1
**228**	248.20	22.00	232.60	51.80	3.84	2.17	0.10	0.00	1
**229**	248.20	22.00	232.60	51.80	3.84	2.17	0.10	0.00	Weighted average of all wild birds
**230**	248.20	22.00	232.60	51.80	3.84	2.17	0.10	0.00	Weighted average of all wild birds
**231**	248.20	22.00	232.60	51.80	3.84	2.17	0.10	0.00	Weighted average of all wild birds
**232**	248.20	22.00	232.60	51.80	3.84	2.17	0.10	0.00	Weighted average of all wild birds
**233**	248.20	22.00	232.60	51.80	3.84	2.17	0.10	0.00	Weighted average of all wild birds
**234**	248.20	22.00	232.60	51.80	3.84	2.17	0.10	0.00	Weighted average of all wild birds
**235**	248.20	22.00	232.60	51.80	3.84	2.17	0.10	0.00	Weighted average of all wild birds

	P (mg)	MG (mg)	K (mg)	NA (mg)	FE (mg)	ZN (mg)	CU (mg)	MN (mg)	Sources
**236**	248.20	22.00	232.60	51.80	3.84	2.17	0.10	0.00	Weighted average of all wild birds
**237**	248.20	22.00	232.60	51.80	3.84	2.17	0.10	0.00	Weighted average of all wild birds
**238**	248.20	22.00	232.60	51.80	3.84	2.17	0.10	0.00	Weighted average of all wild birds
**239**	248.20	22.00	232.60	51.80	3.84	2.17	0.10	0.00	Weighted average of all wild birds
**240**	248.20	22.00	232.60	51.80	3.84	2.17	0.10	0.00	Weighted average of all wild birds
**241**	248.20	22.00	232.60	51.80	3.84	2.17	0.10	0.00	Weighted average of all wild birds
**242**	248.20	22.00	232.60	51.80	3.84	2.17	0.10	0.00	Weighted average of all wild birds
**243**	248.20	22.00	232.60	51.80	3.84	2.17	0.10	0.00	Weighted average of all wild birds
**244**	248.20	22.00	232.60	51.80	3.84	2.17	0.10	0.00	Weighted average of all wild birds
**245**	248.20	22.00	232.60	51.80	3.84	2.17	0.10	0.00	Weighted average of all wild birds
**246**	248.20	22.00	232.60	51.80	3.84	2.17	0.10	0.00	Weighted average of all wild birds
**247**	248.20	22.00	232.60	51.80	3.84	2.17	0.10	0.00	Weighted average of all wild birds
**248**	248.20	22.00	232.60	51.80	3.84	2.17	0.10	0.00	Weighted average of all wild birds
**249**	248.20	22.00	232.60	51.80	3.84	2.17	0.10	0.00	Weighted average of all wild birds
**250**	248.20	22.00	232.60	51.80	3.84	2.17	0.10	0.00	Weighted average of all wild birds

Table 1b. Food categories

Category	Count
Bananas	14
Beans	13
Beverages	2
Bread	9
Bushmeat	22
Domestic meat/poultry	9
Dried seafood	5
Dried freshwater fish	1
Freshwater fish	5
Eggs	2
Fruits	30
Greens	33
Insects	6
Nuts	2
Palm hearts	28
Pasta	2
Rice	3
Seafood	5
Spices	2
Staples	8
Store-bought goods	7
Sugar	4
Vegetables	14
Wild birds	24
Total	250

Table 2. Proxied food items

ID	Variable	Malagasy Name	English Name	Category	Proxy	Proxied nutrients
**3**	fonamb	Fontsy Ambarababoaka	Latundan Banana	Bananas	Traditional banana cooked	All
**4**	fonand	Fontsy andatra	Latundan Banana	Bananas	Traditional banana cooked	All
**8**	fonmak	Fontsy makoa	Red Banana	Bananas	Traditional banana cooked	All
**9**	fonrav	Fontsy malamaravina	Latundan Banana	Bananas	Traditional banana cooked	All
**14**	varfon	Vary sy fontsy	Rice and banana mixture	Bananas	Averaged the nutritional composition of traditional banana and rice, assuming even weights	All
**19**	antsot	Antsotry	Type of beans	Beans	Green bean, cooked	All
**20**	antvong	Antidahivondraka	Type of beans	Beans	Average of cowpeas, kidney, mung, lima beans	All
**21**	bethaz	Betanihazo	Type of beans	Beans	Cowpeas, boiled	All
**22**	grop	Gros pois	Type of beans	Beans	Lima beans	All
**23**	telvo	Telovolana	Type of beans	Beans	Cowpeas, boiled	All
**24**	tsias	Tsiasisa	Type of beans	Beans	Mung beans, boiled, without salt	All
**25**	vam	Vamaho	Black sesames	Beans	Sesame seed, unspecified white or black	All
**26**	voamai	Voamaina	Beans (not specified)	Beans	Kidney beans (mature, boiled, without salt)	All
**27**	voatsi	Voantsiroko	Type of beans	Beans	Cowpeas, boiled	All
**31**	beig	Beignets	Doughnuts	Bread	Wheat, dough, deep fried	All
**32**	belar	Belaro	Type of bread	Bread	English muffin	All
**33**	mofbok	Mofo bokety	Type of bread	Bread	Cassava flour	All
**34**	mofgas	Mofo gasy	Type of bread	Bread	Wheat, dough, deep fried	All
**35**	mofo	Mofo	Bread	Bread	Sourdough bread	All
**36**	mofon	Mofo fontsy	Bread	Bread	Sourdough bread	All

**37**	mofrav	Mofo ravina	Rice and banana bread	Bread	Averaged the nutritional composition of traditional banana and rice, assuming even weights	All
**38**	mofvzah	Mofo vazaha	Flour bread	Bread	Sourdough bread	All
**39**	bokom	Bokombolo	Eastern lesser bamboo lemur	Bushmeat	Wild boar	fat, carbs,fatty acids, chol, fib, suc, vitamins, P, Na
**40**	sokin	Sokina	Greater hedgehog tenrec	Bushmeat	Wild boar	fat, carbs,fatty acids, chol, fib, suc, vitamins, P, Na
**41**	trandr	Trandraka	Tailless tenrec	Bushmeat	Wild boar	fat, carbs,fatty acids, chol, fib, suc, vitamins, P, Na
**42**	varkos	Gidro	White-headed lemur	Bushmeat	Wild boar	fat, carbs,fatty acids, chol, fib, suc, vitamins, P, Na
**43**	ampon	Fotsife	Eastern woolly lemur	Bushmeat	Hapalemur griseus and wild boar	Wild boar: fat, carbs, fib, suc, fatty acids, chol, vitamins, P, Na; Hapalemur: remainder
**44**	andreh	Andrehy	Madagascan rousette	Bushmeat	Pteropus rodricensis and wild boar	Wild boar: fat, carbs, fib, suc, fatty acids, chol, vitamins, P, Na; Pteropus: remainder
**45**	antsaora	Antsaora	Lowland streaked tenrec	Bushmeat	Setifer setosus and wild boar	Wild boar: fat, carbs, fib, suc, fatty acids, chol, vitamins, P, Na; Setifer: remainder

**46**	ayeaye	Aye-aye	Aye-aye	Bushmeat	Eulemur albifrons and wild boar	Wild boar: fat, carbs, fib, suc, fatty acids, chol, vitamins, P, Na; Eulemur: remainder
**47**	babkot	Babakoto	Indri, babakoto, primate	Bushmeat	Hapalemur griseus and wild boar	Wild boar: fat, carbs, fib, suc, fatty acids, chol, vitamins, P, Na; Hapalemur: remainder
**48**	bib	Bibidia	Wildlife (unspecified)	Bushmeat	Eulemur albifrons and wild boar	Wild boar: fat, carbs, fib, suc, fatty acids, chol, vitamins, P, Na; Eulemur: remainder
**49**	falanok	Falanoka	Falanouc, rare mongoose-like mammal, endemic	Bushmeat	Weighted average of bushmeat species	All
**50**	fosa	Fosa	Cat-like, carnivous mammal, endemic	Bushmeat	Weighted average of bushmeat species	All
**51**	jaboa	Jaboady	Small Indian civet	Bushmeat	Weighted average of bushmeat species	All
**52**	kary	Kary	Wild cat	Bushmeat	Weighted average of bushmeat species	All
**53**	lambodia	Lambo dia	Bushpig	Bushmeat	Wild boar	fat, carbs,fatty acids, chol, fib, suc, vitamins, P, Na
**54**	radak	Radaka	Leopard frog	Bushmeat	Frog legs, raw	All
**55**	tomb	Tombokontsodiny	Malagasy civet	Bushmeat	Weighted average of bushmeat species	All
**56**	tongo	Tongo	Red-bellied lemur	Bushmeat	Eulemur albifrons and wild boar	Wild boar: fat, carbs, fib, suc, fatty acids, chol, vitamins, P, Na; Eulemur: remainder

**57**	tsid	Tsidy	Brown mouse lemur	Bushmeat	Eulemur albifrons and wild boar	Wild boar: fat, carbs, fib, suc, fatty acids, chol, vitamins, P, Na; Eulemur: remainder
**58**	tsits	Tsitsiha	Greater dwarf lemur	Bushmeat	Eulemur albifrons and wild boar	Wild boar: fat, carbs, fib, suc, fatty acids, chol, vitamins, P, Na; Eulemur: remainder
**59**	varkan	Varikandavaka	Lepilemur sp.	Bushmeat	Hapalemur griseus and wild boar	Wild boar: fat, carbs, fib, suc, fatty acids, chol, vitamins, P, Na; Hapalemur: remainder
**60**	vontsi	Vontsira	Ring-tailed mongoose	Bushmeat	Weighted average of bushmeat species	All
**64**	angozy	Angozy	Zebu skin and fat (indicine cattle, humped cattle, Brahman)	Domestic meat/poultry; pork/beef	Beef, subcutaneous fat, cooked	All
**72**	ankor	Anankorana	Dried white shrimp	Dried seafood	(Palaemon spp; Penaeus sp.)	All
**74**	patsa	Patsa	Dried red shrimp	Dried seafood	(Palaemon spp; Penaeus sp.)	All
**75**	lksir	Laoko sira	Salted fish (typically larger fish)	Dried seafood	Dried saltwater fish	All
**97**	zano	Zano	Custard apple	Fruits	Weighted average of all fruits	D, E, B6, fol, P, Mn
**99**	mangvz	Mangambazaha	Type of fruit	Fruits	Mango	All
**100**	matbar	Matohabaratra	Type of fruit	Fruits	Weighted average of all fruits	All
**101**	sakoan	Sakoana	Plum of Cythera, apple kythira, tree Kythera	Fruits	Weighted average of all fruits	All

**102**	tomat	Tomate	Tomato, ripe	Fruits	Fresh, red, roma tomato	All
**103**	tsamkob	Tsaminakoba	Type of fruit	Fruits	Weighted average of all fruits	All
**104**	vanok	Vanonoka	Type of fruit	Fruits	Weighted average of all fruits	All
**105**	vaor	Vaory	Type of fruit	Fruits	Weighted average of all fruits	All
**106**	voafk	Voafoko	Type of fruit	Fruits	Weighted average of all fruits	All
**108**	voakz	Voankazo	Fruit (not specified)	Fruits	Weighted average of all fruits	All
**109**	voasir	Voasirindrina	Type of fruit	Fruits	Weighted average of all fruits	All
**110**	vong	Vongo	Type of fruit	Fruits	Weighted average of all fruits	All
**111**	vontsik	Vontsikobila	Type of fruit	Fruits	Weighted average of all fruits	All
**125**	anab	Anambe	Type of greens	Greens	Bok choy, raw	All
**126**	anan	Anana	General term for greens	Greens	Weighted average of all greens	All
**127**	anbaml	Anamabala	Type of greens	Greens	Weighted average of all greens	All
**128**	anbon	Ananabontro	Type of greens	Greens	Weighted average of all greens	All
**129**	angid	Anangidy	Type of greens	Greens	Weighted average of all greens	All
**130**	angis	Anangisa	Type of greens	Greens	Weighted average of all greens	All
**131**	anjf	Ananjofo	Type of greens	Greens	Weighted average of all greens	All
**132**	anlao	Anamafana	Toothache plant, jambu, spot plant	Greens	Weighted average of all greens	All
**133**	anpats	Anampatsy	Type of greens	Greens	Weighted average of all greens	All
**134**	antarik	Anantarika	Type of greens	Greens	Weighted average of all greens	All
**135**	antsal	Anantsalepo	Type of greens	Greens	Weighted average of all greens	All
**136**	antsek	Anantsenko	Wild ferns	Greens	Fiddlehead ferns	All
**137**	antsid	Anantsindra	White nightshade	Greens	Nightshade, Ethiopian (Solanum aethiopicum), fruit	All
**138**	antsinah	Anantsinahy	Type of greens	Greens	Weighted average of all greens	All
**139**	antsir	Anantsiriry	Rice paddy clovers	Greens	Weighted average of all greens	All
**140**	antsom	Anantsonga	Type of greens	Greens	Weighted average of all greens	All

**141**	fela	Felagna	Flowers from Hesikesika	Greens	Weighted average of all greens	All
**142**	pets	Petsay	Turnip greens	Greens	Brassica rapa: Turnip greens, raw	All
**143**	ravhets	Ravina hetsika	Type of greens	Greens	Weighted average of all greens	All
**144**	raving	Ravina angivy	Type of greens	Greens	Weighted average of all greens	All
**145**	solof	Solofoko	Type of greens (like hesikesika but wild forest version)	Greens	Weighted average of all greens	All
**147**	ankantel	Ankaninantely	Fresh honeycomb	Insects	Honey, raw	All
**148**	lafa	Lafa	Beetle larvae	Insects	Beetle larvae (mealworm)	All
**149**	lafbit	Lafa bitay	Rhinoceros beetle grub	Insects	Beetle larvae (mealworm)	All
**150**	lafoh	Lafa fohy	Beetle larvae	Insects	Beetle larvae (mealworm)	All
**151**	tsibo	Tsibobona	No common name	Insects	Cybister sp.	All
**153**	vakor	Vakoromanga	Type of nut	Nuts	Peanuts, all types, boiled, with salt	All
**182**	longf	Longo fy	Type of pasta	Pasta	Pasta, homemade, with egg, cooked	All
**183**	pate	Pate	Pasta	Pasta	Pasta, homemade, with egg, cooked	All
**184**	varh	Vary horaka	Paddy rice	Rice	Brown rice, boiled	All
**185**	varj	Vary jinja	Swidden rice	Rice	Brown rice, boiled	All
**186**	vary_mas	Vary masaka	Cooked rice	Rice	Brown rice, boiled	All
**188**	ordv	Orandava	Prawns	Seafood	Shrimp, combined with weighted average of all seafood	fatty acids; chol; A, E, fol, B12; Mg, K, Na, Fe, Zn, Cu, Mn, B6
**189**	orita	Orita	Octopus	Seafood	Shrimp, combined with weighted average of all seafood	fatty acids; chol; A, E, fol, B12; Mg, K, Na, Fe, Zn, Cu, Mn, B6

**190**	orlen	Orana legny	Fresh shrimp	Seafood	Weighted average of all seafood	Cu, Mn, B6
**200**	hanbrk	Hanimbiroka	Unspecified type of root vegetable	Staples	Root vegetable average	All
**214**	agiv	Angivy	African eggplant, type of nightshade	Vegetables	Nightshade, Ethiopian (Solanum aethiopicum), fruit	All
**219**	papang	Papangay	Loofa gourd, sponge gourd	Vegetables	Weighted average of all vegetables	fatty acids, suc, D, E, C B6, fol, Mg, Zn, Cu, Mn
**220**	patsol	Patrola	Loofa gourd, sponge gourd	Vegetables	Weighted average of all vegetables	fatty acids, suc, D, E, C B6, fol, Mg, Zn, Cu, Mn
**225**	ambarbanj	Ambarabanjina	Type of vegetable in cucumber family	Vegetables	Cucumber, with peel, raw	All
**226**	voangar			Vegetables	Chayote, boiled without salt	All
**228**	akanga	Akanga	Guinea Fowl	Wild birds	Guinea hen, meat and skin	All
**229**	ambos	Ambosanga	Type of wild bird	Wild birds	Average of dove, wild duck, pheasant, quail, pigeon	All
**230**	boeza	Boeza	Lesser vasa parrot	Wild birds	Average of dove, wild duck, pheasant, quail, pigeon	All
**231**	dom	Domohina	Type of wild bird	Wild birds	Average of dove, wild duck, pheasant, quail, pigeon	All
**232**	fangad	Fangadiovy	Type of wild bird	Wild birds	Average of dove, wild duck, pheasant, quail, pigeon	All
**233**	firas	Firasa	Hawk	Wild birds	Average of dove, wild duck, pheasant, quail, pigeon	All
**234**	fod	Fody	Type of wild bird	Wild birds	Average of dove, wild duck, pheasant, quail, pigeon	All

**235**	hitsik	Hetsiketsika	Hawk	Wild birds	Average of dove, wild duck, pheasant, quail, pigeon	All
**236**	kaikmav	Kaikimavo	Type of wild bird	Wild birds	Average of dove, wild duck, pheasant, quail, pigeon	All
**237**	kilan	Kilandy	Type of wild bird	Wild birds	Average of dove, wild duck, pheasant, quail, pigeon	All
**238**	koa	Koa	Type of wild bird	Wild birds	Average of dove, wild duck, pheasant, quail, pigeon	All
**239**	lampir	Lampirana	Type of wild bird	Wild birds	Average of dove, wild duck, pheasant, quail, pigeon	All
**240**	mariah	Mariha	Type of wild bird	Wild birds	Average of dove, wild duck, pheasant, quail, pigeon	All
**241**	papa	Papango	Hawk	Wild birds	Average of dove, wild duck, pheasant, quail, pigeon	All
**242**	sob	Sobery	Type of wild bird	Wild birds	Average of dove, wild duck, pheasant, quail, pigeon	All
**243**	totor	Totoroka	Type of owl	Wild birds	Average of dove, wild duck, pheasant, quail, pigeon	All
**244**	triala	Tritrikiala	Type of wild bird	Wild birds	Average of dove, wild duck, pheasant, quail, pigeon	All
**245**	tsik	Tsikoza	Type of wild bird	Wild birds	Average of dove, wild duck, pheasant, quail, pigeon	All
**246**	tsir	Tsiriry	Type of wild bird	Wild birds	Average of dove, wild duck, pheasant, quail, pigeon	All
**247**	vorad	Vorona dy	Type of wild bird	Wild birds	Average of dove, wild duck, pheasant, quail, pigeon	All
**248**	vorb	Vorombe	Hawk	Wild birds	Average of dove, wild duck, pheasant, quail, pigeon	All
**249**	vorkah	Voronkahaka	Type of wild bird	Wild birds	Average of dove, wild duck, pheasant, quail, pigeon	All
**250**	vorts	Vorontsaina	Type of wild bird	Wild birds	Average of dove, wild duck, pheasant, quail, pigeon	All

Table 3. References

	**References**	**Number of citations**
**1**	US Department of Agriculture, Agricultural Research Service, Nutrient Data Laboratory. USDA National Nutrient Database for Standard Reference, Release 28.	101
**2**	Lukmanji, Z., E. Hertzmark, N. Mlingi, V. Assey, G. Ndossi, and W. Fawzi. 2008. Tanzania food composition tables. Muhimbili University of Health and Allied Sciences, Tanzania Food and Nutrition Center, Harvard School of Public Health, First Edition.	59
**3**	Korkalo, L., H. Hauta-alus, and M. Mutanen. 2011. Food composition tables for Mozambique. Department of Food and Environmental Sciences, University of Helsinki, Finland	26
**4**	Laboratory analysis*	20
**5**	Stadlmayr et al. 2010. Composition of selected foods from West Africa. Food and Agriculture Organization of the United Nations	16
**6**	Food Plant Solutions Rotarian Action Group. Potentially important food plants of Madagascar. Food Plant Solutions Field Guide - Madagascar, Version 1, September 2015	15
**7**	Food Standards Australia. 2010. Nutrient tables for use in Australia (NUTTAB)	12
**8**	Institute of Nutrition, Mahidol University (2014). ASEAN Food Composition Database, Electronic version 1, February 2014, Thailand.	10
**9**	Stadlmayr et al. 2012. West African food composition table. Food and Agriculture Organization of the United Nations, Rome.	10
**10**	Finke, M.D. 2004. Nutrient content of insects. Encyclopedia of Entomology. Scottsdale, Arizona, USA	4
**11**	Menchu, M.T., and H. Mendez. 2007. Tabla de composicion de alimentos de Centroamerica. INCAP 2nd Edition.	3
**12**	Food and Agriculture Organization of the United Nations. 1972. Food composition table for use in East Africa. US Department of Health, Education, and Welfare.	2
**13**	Hotz, C., A. Lubowa, C. Sison, M. Moursi, and C. Loechl. 2012. A food composition table for central and eastern Uganda. HarvestPlus, Washington DC.	2
**14**	Kunchit, J., P. Puwastien, A. Nitithamyong, P. Sridonpai, A. Somjai. Institute of Nutrition, Mahidol University 2015. Thai Food Composition Database, Online version 1, January 2016, Thailand.	2
**15**	Banjo, A.D., Lawal, O.A., and E. A. Songonuga. 2006. The nutritional value of fourteen species of edible insects in southwestern Nigeria. African Journal of Biotechnology 5(3): 298-301.	1
**16**	Bilgin Ş and ZUC Fidanbaş, 2011. Nutritional properties of crab (Potamon potamios Olivier, 1804) in the lake of Eğirdir (Turkey). Pak Vet J, 31(3): 239-243.	1

**17**	Edem, D.O., O.U. Eka, and E.T. Ifon. 1984. Chemical evaluation of the nutritive value of the raffia palm fruit. Food Chemistry 15(1): 9-17	1
**18**	Gobble, R., M. Taylor, and G. Lyons. 2010. Factsheet no. 10 chilli leaf. Australian Centre for International Agricultural Research.	1

* Mineral analysis was conducted in the Glahn laboratory at Cornell University following an established methodology (Tako et al. 2013).

Tako, E, Hoekenga, OA, Kochian, LV, & Glahn, RP (2013). High Bioavailablilty Iron Maize (zea maysl.) Developed Through Molecular Breeding Provides More Absorbable Iron In-vitro (caco-2) And In-vivo (gallusgallus). *Annals of Nutrition and Metabolism*, *63*, 252.

Supplemental Table S4: Summary statistics for food categories (g/consumed/day per individual, at household meals)

Food category	Mean (g)	SD (g)	Min (g)	Max (g)
Cereals	624.8	333.5	39.3	2134.8
Roots and tubers	137.6	248.4	0.0	3571.7
Vegetables	79.3	63.9	0.0	705.1
Fruits	144.8	172.7	0.0	2246.8
Wild meat	5.5	16.4	0.0	234.5
Domestic meat	32.0	44.9	0.0	544.2
Eggs	0.1	1.2	0.0	24.5
Fish	16.9	25.6	0.0	653.2
Pulses	4.8	10.1	0.0	154.2
Milk and dairy	1.6	11.1	0.0	262.1
Oils/fats	2.7	3.7	0.0	37.1
Sugar	36.3	138.5	0.0	3044.5
Miscellaneous	8.6	101.2	0.0	3801.2

Supplemental Table S5: Dietary intake of carbohydrates, fats, and other nutrients without an Estimated Average Requirement provided by the Institutes of Medicine

Nutrient	Unit (person/day)	Mean	Median	SD	Min	Max
Carbohydrates	g	262.8	257.1	138.9	25.0	1246.1
Total fat	g	30.8	27.1	21.6	2.0	268.5
Saturated fat	g	21.2	19.6	12.6	1.5	88.6
Monounsaturated fat	g	7.0	4.8	8.9	0.2	172.8
Polyunsaturated fat	g	2.7	2.4	2.2	0.2	33.6
Cholesterol	mg	50.8	32.6	58.6	0.0	827.8
Fiber	g	18.7	15.8	14.0	1.5	166.6
Sucrose	g	18.6	12.2	20.4	0.3	331.6
Potassium	mg	2552.5	1914.7	2259.8	144.8	27628.3
Manganese	mg	2.8	2.7	1.5	0.2	12.6

FIGURES

Supplemental Fig. S1: The linear relationship between increasing vitamin B12 consumption and increasing variability in vitamin B12 consumption. Note: 60/623 (9.6%) observations with greater than 100% monthly sufficiency are not shown, for visualization purposes. This relationship is consistent across most nutrients we analyzed.

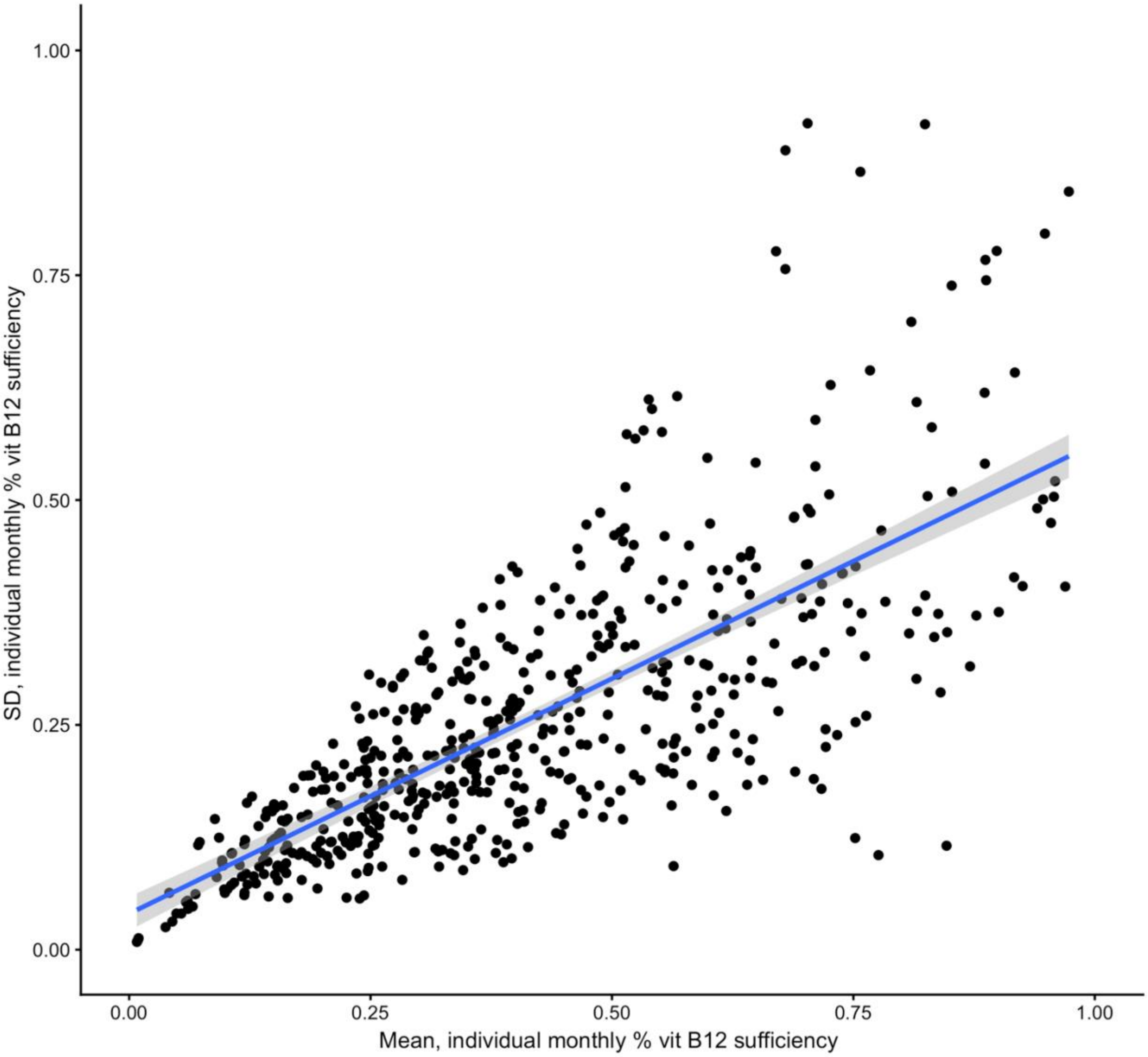

Supplemental Fig. S2: Consumption smoothing

Village 1

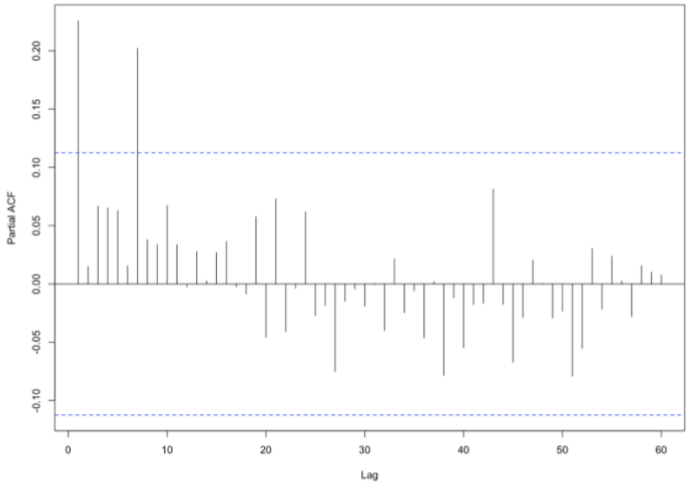

Village 2

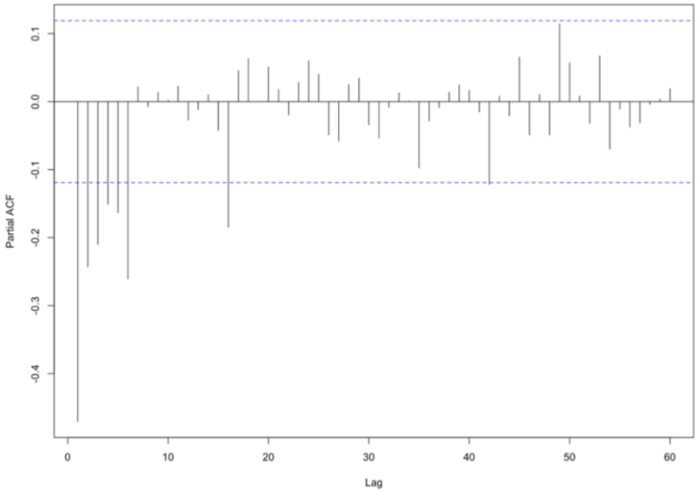

Kilocalorie correlograms for each village, based on fitting autoregressive integrated moving average (ARIMA) models. Blue lines indicate 95% confidence intervals significance intervals; any correlation coefficient within blue lines is insignificant). The village 1 series is stationary in original form; the village 2 series was first-differenced for stationarity. An ARIMA(1,0,2) model provided best fit for village 1; an ARIMA(0,1,1) provided best fit for village 2. Village 1 shows no autocorrelation; the kilocalorie series approximates a random walk. Village 2 shows negative correlation between a given day and the subsequent 1-6 days, suggesting that households mildly smooth consumption over this time frame.

Supplemental Fig. S3: The distribution of Household Dietary Diversity Scores across all household days (panel A) and their seasonal variability (panel B)

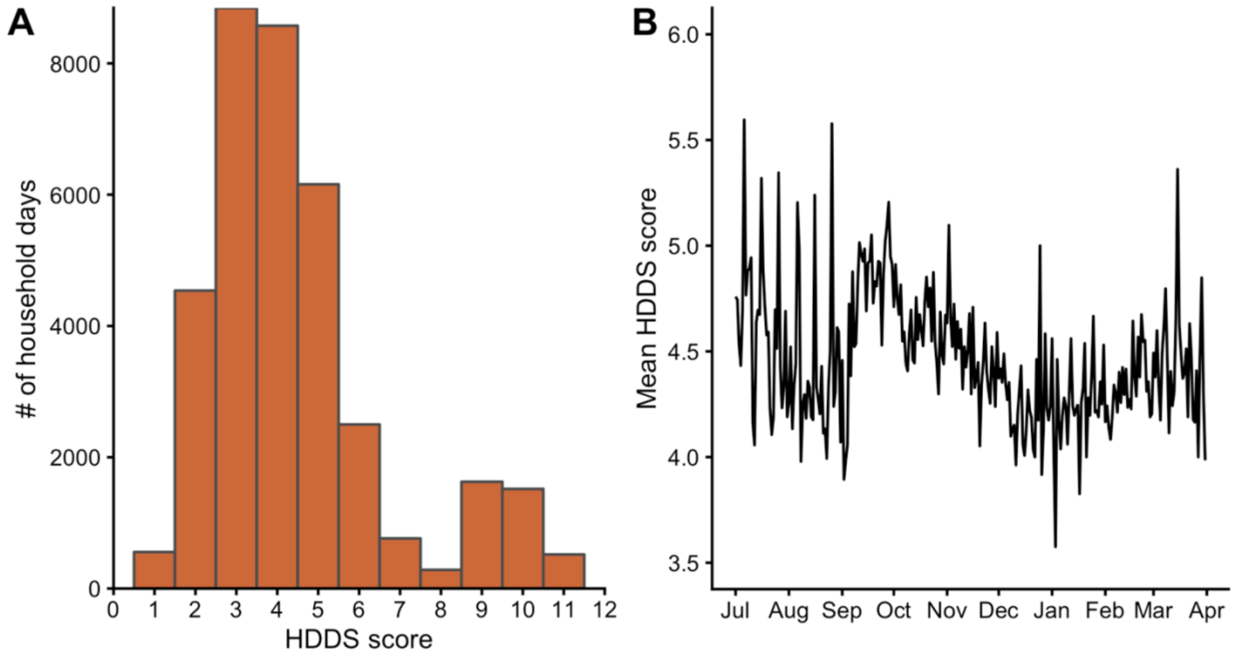

Supplemental Fig. S4: The distribution of Food Consumption Scores for the full set of households weekly experiences (n=5,340)

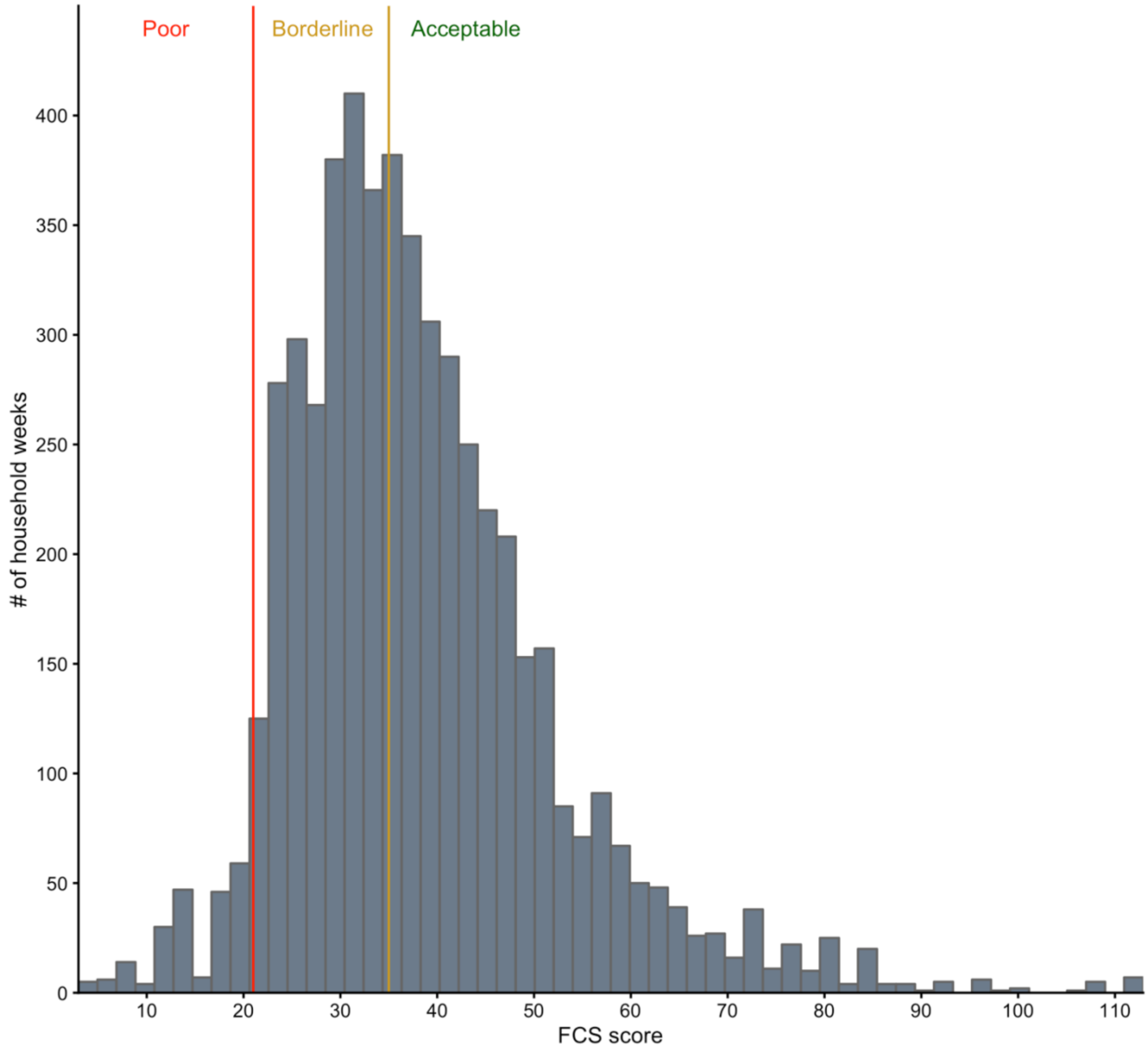

The FCS ranges from 0 to 112. Scores ≤21 are considered "poor," scores between 21.5 and 35 are "borderline," (BL) and scores >35 "acceptable."

Supplemental Fig. S5: The distribution of Minimum Dietary Diversity Scores for Women (MDD-W) for the full set of reproductive-aged women's daily experiences (n=36,662)

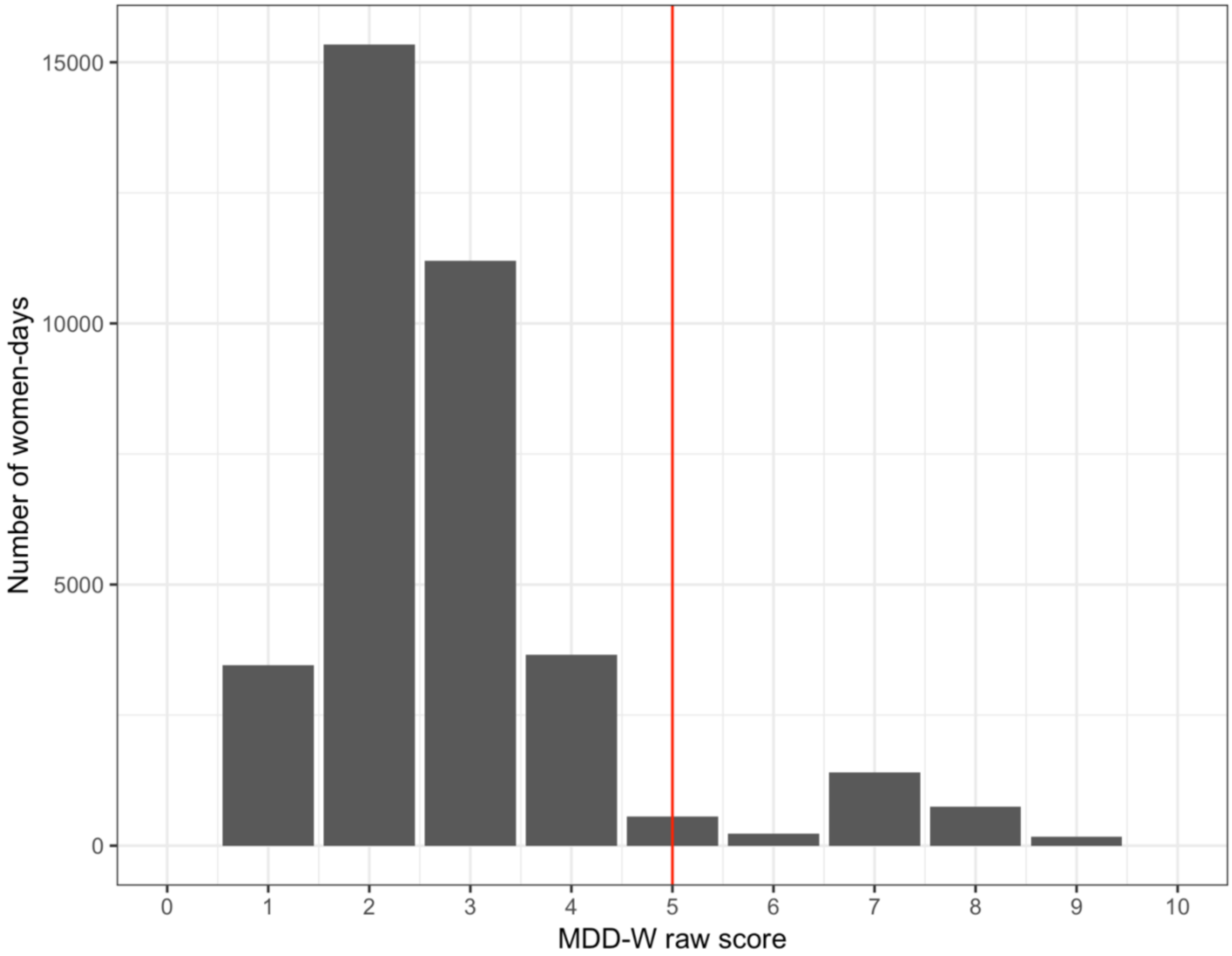

The MDD-W ranges from 0 to 10 with five food groups being the threshold for high dietary diversity. Anything less than five food groups indicates low dietary diversity.
